# Pharmacokinetics of a Single Dose of Turmeric Curcuminoids Depends on Formulation: Results of a Human Crossover Study

**DOI:** 10.1093/jn/nxab087

**Published:** 2021-04-20

**Authors:** Pascale Fança-Berthon, Mathieu Tenon, Sabrina Le Bouter-Banon, Alexis Manfré, Corinne Maudet, Angelina Dion, Hélène Chevallier, Julie Laval, Richard B van Breemen

**Affiliations:** Naturex SA, Avignon, France; Naturex SA, Avignon, France; Biofortis SAS, Saint Herblain, France; Naturex SA, Avignon, France; Biofortis SAS, Saint Herblain, France; Biofortis SAS, Saint Herblain, France; Biofortis SAS, Saint Herblain, France; Naturex SA, Avignon, France; Linus Pauling Institute, Oregon State University, Corvallis, OR, USA

**Keywords:** absorption, *Curcuma longa*, curcumin, metabolism, relative bioavailability, metabolites, curcuminoids

## Abstract

**Background:**

Curcuminoids from turmeric rhizome have significant health benefits but low bioavailability.

**Objectives:**

To assess the pharmacokinetics of a novel natural turmeric dried colloidal suspension compared with 4 other turmeric formulations (including a standardized extract) at their respective recommended dosages.

**Methods:**

Thirty healthy men and women (18 to 45 y old) were enrolled in a randomized, open-labeled, crossover trial, and sequentially consumed single oral doses of standard turmeric extract (1500 mg), liquid micellar preparation (1000 mg), piperine-curcuminoid combination (1515 mg), phytosome formulation (1000 mg), or the dried colloidal suspension (300 mg). Eleven blood samples were obtained over 24 h, plasma was extracted with or without deconjugation with β-glucuronidase or sulfatase, and ultra-high-pressure liquid chromatography/tandem MS was used to quantify the 3 parent curcuminoids and 12 metabolites. Classical pharmacokinetics parameters were derived.

**Results:**

The total AUC values of unconjugated curcuminoids were highly variable within participants, with no significant differences between formulations. However, the AUC values for total curcuminoids (including all metabolites) showed significant product effects. Indeed, the micellar preparation delivered higher levels of total curcuminoids than any other formulation (8540 ng·h/mL), reaching significance when compared with the dried colloidal suspension and standard extract (6520 and 5080 ng·h/mL, respectively). After dose normalization, both micellar and dried colloidal formulations showed significantly higher AUC levels than the standard extract (respectively 136 and 72.9, compared with 3.7 ng·h/mL/mg). Total curcuminoid absorption levels were also significantly higher for the dried colloidal suspension when compared with either piperine or phytosome formulations. Interestingly, no significant differences were observed between the piperine-curcuminoid combination and the standard extract. No serious adverse events were reported.

**Conclusions:**

The administration of a low dose of the novel natural dried colloidal suspension provided high unconjugated and conjugated curcuminoid absorption, with significant beneficial differences when compared with the high dose of standard extract.

This trial was registered at clinicaltrials.gov as NCT03621865.

## Introduction

The rhizome of *Curcuma longa* (turmeric) is used as a spice, as a traditional medicinal in Asia (1), and also as one of the most popular botanical dietary supplements in the United States (2). Supported by both in vitro and in vivo data, turmeric has antioxidant and anti-inflammatory activities, can stimulate the immune system, and has anticancer activity (3–9). The curcuminoids curcumin, demethoxycurcumin (DMC), and bisdemethoxycurcumin (BDMC) are viewed as the most bioactive turmeric constituents associated with its potential health benefits.

**FIGURE 1:**
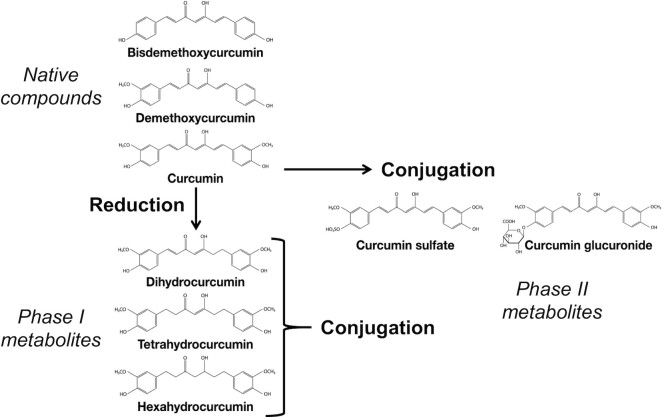
Chemical structures of curcuminoids from turmeric extract and principal phase I and phase II metabolites.

Orally administered turmeric native compounds have a low intestinal absorption (10–12), and most are excreted unchanged in the feces (13). This observation is consistent with the poor absorption (0–20%) predicted by the Caco-2 human intestinal epithelial monolayer absorption model (14). Phase I reduction and phase II conjugation (**Figure 1**) appear to be the primary metabolic pathways of absorbed curcuminoids, taking place in the intestinal and hepatic tissues (15–18). In addition to rapid metabolism, absorbed curcumin is rapidly eliminated from the systemic circulation (5, 12, 15). Curcumin can also be metabolized by an NADPH-dependent curcumin/dihydrocurcumin reductase in intestinal microbiota to form dihydrocurcumin (DHC) and tetrahydrocurcumin (THC) (19). THC but not curcumin have been reported to accumulate in rat tissues, suggesting that microbiota could be also a factor in the metabolism and bioavailability of curcuminoids (20).

Curcuminoids are hydrophobic with high solubility in organic solvents (10) but much lower solubility in water. For example, the solubility of curcumin in aqueous buffer (pH 5.0) is only 11 ng/mL (21). As described by FAO in 2004, curcumin also exhibits keto–enol tautomerism, with the favored keto form being stable under acid conditions but insoluble in water, whereas its enol form is soluble in water but unstable above pH 7 (21, 22).

Due to the combination of poor oral absorption, rapid metabolism, and rapid elimination from the systemic circulation, systemic concentrations of unconjugated curcumin do not exceed the low micromolar concentration in humans even after oral doses ≤12 g. To enhance the oral bioavailability of curcumin in turmeric extracts, a variety of formulations have been developed and are marketed to consumers. These formulations include liposomal curcumin, nanoparticles containing curcumin, and adjuvants like piperine and phospholipids (15, 17, 23). Unfortunately, few of these formulations have been tested in clinical trials using clinically relevant doses in comparison with a standard turmeric extract containing 95% curcuminoids.

The aim of this study was to assess the bioavailability of 5 formulations of turmeric extract at clinically relevant and recommended dosages for health conditions such as inflammation, joint health, cardiometabolic health, liver health, and cognition (24–31). The pharmacokinetics of curcuminoids in a standardized turmeric extract were compared with a combination containing the adjuvant piperine (inhibits phase II metabolism) (32), and 3 alternatives designed to enhance absorption including a phytosome formulation (33), a liquid micellar preparation (34), and a new dried colloidal suspension. An analytical method was also developed to quantify separately the 3 major turmeric curcuminoids and their phase I and II metabolites in human plasma to support pharmacokinetics modeling and to clarify routes of metabolism.

## Methods

### Ethics

The protocol and all the documents of the trial were approved by the Committee for Patient Protection (Comité de Protection des Personnes; Ouest V, Rennes, France; reference number 18/050-1) and by the National Agency for Drug Safety (Agence national de sécurité du medicament; registration number 2018_A01390-55). Written informed consent was obtained for all the participants before inclusion in the study.

### Study materials

All turmeric supplements for human consumption were encapsulated in hydroxypropyl methylcellulose capsules. The reference product was a standard turmeric powder extract (STE) containing 95% curcuminoids (Naturex) and tested at the recommended efficacy dosage of 1500 mg (1425 mg curcuminoids) in 4 capsules. A phytosome formulation (PHYT) of turmeric extract, phosphatidyl choline, and microcrystalline cellulose (Meriva; Indena) containing 18–22% curcuminoids was administered at a dosage of 1000 mg in 2 capsules (180–220 mg curcuminoids) as recommended by the manufacturer. A dried colloidal suspension (TPG) of standard turmeric extract, quillaja extract, sunflower oil, and acacia gum containing 30% curcuminoids (Turmipure GOLD; Naturex) was tested at a dosage of 300 mg (90 mg curcuminoids) in 1 capsule as directed. A turmeric extract standardized to 95% curcuminoids (C3 complex; Sabinsa) at 1500 mg (1425 mg curcuminoids) combined with 15 mg of a pepper extract standardized to 95% piperine (Bioperine; Sabinsa) was administered in 3 capsules (TEP). A liquid micellar preparation (NOV) containing 6% curcuminoids (NovaSOL; Aquanova AG) was administered at a dosage of 1000 mg (60 mg curcuminoids) in 2 capsules. Ten capsules of each turmeric product were analyzed using ultra-high-pressure liquid chromatography (UHPLC)/tandem MS as described in **Supplemental Methods** to ascertain the actual quantities of curcuminoids administered (**Table 1**).

**TABLE 1:** Curcuminoid analysis of a single dose of the turmeric formulations STE, TEP, PHYT, NOV, and TPG administered to participants^1^

Amount, mg	STE	TEP	NOV	PHYT	TPG
Curcumin	1140 ± 35.5	1120 ± 30.2	50.2 ± 1.64	146 ± 4.93	74.3 ± 2.26
DMC	213 ± 7.52	230 ± 6.94	10.9 ± 0.38	28.8 ± 1.07	13.9 ± 0.50
BDMC	19.7 ± 0.89	28.5 ± 0.84	1.54 ± 0.07	3.85 ± 0.24	1.29 ± 0.05
Total (measured)	1380 ± 43.6	1380 ± 37.5	62.7 ± 2.07	179 ± 6.19	89.4 ± 2.74
Total (product label)	1425	1425	60	180–220	90

1 Values are means ± SD from the quantification using HPLC-UV of 10 individual capsules and are reported based on the number of capsules ingested by participants for each formulation: STE: 4 capsules; TEP: 3 capsules; NOV: 2 capsules; PHYT: 2 capsules; TPG: 1 capsule. See Supplemental Methods for details. BDMC, bisdemethoxycurcumin; DMC, demethoxycurcumin; HPLC-UV, high-pressure liquid chromatography with ultra-violet spectroscopy; NOV, liquid micellar formulation; PHYT, phytosome formulation; STE, standard turmeric extract; TEP, piperine-curcuminoids combination; TPG, Turmipure Gold formulation.

### Study design

Volunteers were screened until 30 adult participants were enrolled in a randomized, open-labeled, crossover study following guidelines for appropriate sample size selection in pilot studies (35–40). In chronological order of inclusion, participants were assigned a sequence of turmeric products. Randomization was carried using SAS software version 9.3 (SAS Institute) and a Latin square design. Inclusion and exclusion criteria are detailed in **Supplemental Table 1**. In addition, to ensure the health status of the volunteers, blood chemistry was tested at the screening visit (glycemia, alanine aminotransferase, aspartate aminotransferase, γ-glutamyl transpeptidase, urea, creatinine, and complete blood count) and pregnancy was tested for nonmenopausal women (β-HCG, Beta human chorionic gonadotropin).

Participants were recruited through the Biofortis Clinical Investigation Unit (Saint-Herblain, France) from July 2018 to October 2018. The first of the 5 intervention visits, taking place ≤3 wk after the screening visit, also constituted the randomization visit. Each experimental session was separated by 7 to 14 d. Volunteers were not allowed to consume curcumin-containing food supplements or foods (turmeric, curry, etc.) for 2 wk prior to testing and during the study, and had to keep their life habits (eating, physical activity, tobacco and alcohol consumption) stable during the entire study. Taking new medications, especially antibiotics, laxatives, antidiarrheal therapy, or medications or food supplements containing plant extracts, vitamins, and minerals, was not permitted during the study, except in case of extreme necessity. Participants were also asked to avoid consuming any medication (e.g., paracetamol or ibuprofen) within 24 h prior to each experimental session, not to have a heavy meal or alcohol abuse, nor strenuous physical exercise within 48 h prior to each experimental session, and not to smoke the morning of each experimental session. Finally, participants were asked to keep the same mode of transportation to come to the investigation site.

**FIGURE 2:**
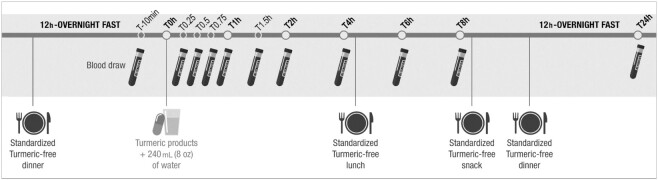
Schematic representation of study procedures. Participants were provided turmeric-free dinner the night before through home delivery. After a 12-h overnight fast, they were welcomed at the study site and prepared for blood draw and product consumption. Participants were asked to stay at the study site during the day and were provided turmeric-free lunch (after the 4-h time point) and snack (after the 8-h time point) before being allowed to leave. Water was not permitted 1 h before or after product administration but was allowed at all other times ad libitum. The last turmeric-free dinner, before the 24-h time point, was provided through home delivery. After a 12-h overnight fast, participants were asked to come back for a last blood draw corresponding to the last time point (24 h).

Turmeric-free meals and snacks were provided to each participant beginning the night before and during each experimental session (meal compositions are detailed in **Supplemental Table 2**). After a 12-h overnight fast, vital signs were measured prior to time of blood sampling (see study procedures in **Figure 2**). At each session, any side effects and potential concomitant treatments were recorded.

### Sample collection and preparation

Blood was drawn through a catheter in one of the forearm veins. A baseline blood sample was obtained (T − 10 min), followed by the consumption of 1 of the 5 products with 240 mL water (T0). Additional blood samples were drawn at 0.25, 0.50, 0.75, 1.00, 1.50, 2.00, 4.00, 6.00, 8.00, and 24.00 h following product consumption (Figure 2). Blood samples (5 mL each) were collected in tubes containing sodium citrate 3.8% (Greiner bio-one), centrifuged at 2200 × *g* for 15 min at 4°C, and the plasma was aliquoted into microtubes. Plasma samples were stored at −80°C until analysis. Water was not permitted 1 h before or after product administration but was allowed at all other times ad libitum.

### Extraction of curcuminoids

For deconjugation, plasma (100 μL) was incubated with β-glucuronidase (2500 U; #G8295; Merck) in phosphate buffer (100 mM, pH 6.8, 37°C), or sulfatase (200 U; #S9626; Merck) in acetate buffer (0.1 M, pH 5.0, 37°C) for 1 h with constant mixing. Protein precipitation, solid-phase extraction, and filtration was carried out using Captiva EMR-Lipid 96-well plates (Agilent Technologies) as explained in Supplemental Methods.

### UHPLC-tandem MS of curcuminoids in plasma

Unconjugated and conjugated curcumin, DMC, BDMC, THC, and HHC concentrations were measured in plasma using UHPLC-tandem MS on an Agilent 6420 triple quadrupole mass spectrometer equipped with a 1290 UHPLC system and electrospray (detailed procedures in Supplemental Methods and **Supplemental Tables 3** and **4**).

### Trial hypotheses and statistical analyses

The study primary end point was the dose-normalized AUC of total plasma curcuminoids (curcumin, DMC, BDMC, plus their metabolites) from 0 to 24 h (AUC 0–24 h/dose; expressed in ng·h/mL/mg). Our hypothesis was that the dose-normalized AUC of total plasma curcuminoids for the colloidal suspension TPG would be higher than that observed for the standard extract STE. Secondary outcomes included peak plasma concentration (Cmax), dose-normalized Cmax, time to maximum concentration (Tmax), half-life (*t*_1/2_), terminal elimination rate constant (λz), AUC 0–24h,  and AUC 0–8h. The total AUC values were determined for plasma-specific major curcuminoids and metabolites as well as for total curcuminoids (see Supplemental Methods for a detailed description of the statistical methods used and details on the mixed models).

Statistical analyses were performed using SAS software version 9.3 (SAS Institute) on the intent-to-treat (ITT) population (including all randomly assigned participants in the study, who consumed each study product regarding their randomization sequence, *n* = 30). Because similar results were observed on the per-protocol population (participants who presented no major protocol deviations, *n* = 29) the corresponding analyses are not presented here. Pharmacokinetic parameters were calculated using R software version 3.5.1 (ncappc package, noncompartmental method) for all tested formulations. Results are expressed as observed mean ± SD. Analyses were performed on log-transformed data to preserve study end-point normality and/or homoscedasticity (assumptions of normality and homoscedasticity for linear models were investigated by graphical representations of residuals produced by statistical models), and multiple pairwise comparisons between products were performed only if a significant product effect was observed. In this case, Tukey adjustment was used to calculate adjusted *P* values from the models, and the level of significance was taken to be *P* < 0.05. In regard to the primary outcome of the study and for clarity, multiple comparisons shown in this manuscript are using either the novel dried colloidal suspension TPG or the reference standard extract STE as main comparatives.

## Results

### Participant demographics and study materials

**FIGURE 3:**
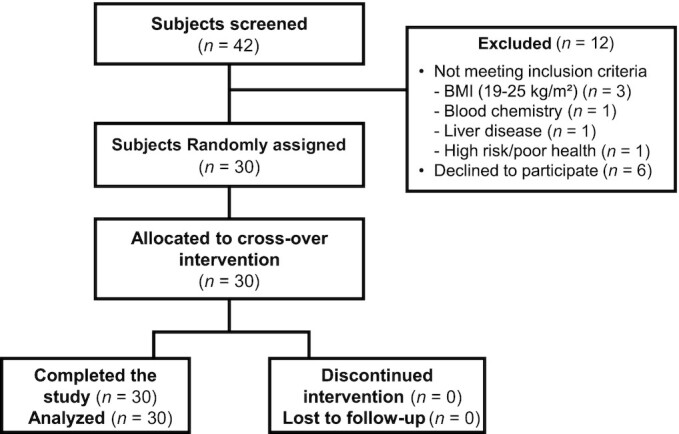
Clinical study CONSORT diagram. A total of 42 volunteers were screened, of which 12 did not meet the inclusion/exclusion criteria. Thirty participants were enrolled and completed the study. No participants discontinued the intervention, were lost to follow-up, or were excluded from the data analysis. No serious adverse events were reported.

Forty-two volunteers were screened, 6 declined to participate, 6 were disqualified due to health considerations, and 30 were enrolled in the study (**Figure 3**). All 30 of the participants (14 men and 16 women) completed the study, and no serious adverse events were reported after randomization. The mean age of the participants was 33.6 ± 6.8 y, the mean BMI was 22.1 ± 2.1 kg/m^2^, and all blood chemistry and physical examination values were normal (**Table 2**). The daily dosages of specific curcuminoids in each formulation were determined using UHPLC-tandem MS. In all cases, the total curcuminoid concentrations were in accordance with the nominal values reported on the product labels (Table 1).

**TABLE 2:** Demographics and biological data of study participants at screening^1^

	All participants (*n* = 30)	Men (*n* = 14)	Women (*n* = 16)
Age, y	33.6 ± 6.79	36.7 ± 5.50	30.8 ± 6.75
Height, cm	170 ± 10.3	179 ± 5.15	163 ± 8.16
Weight, kg	64.5 ± 11.1	72.8 ± 8.33	57.3 ± 7.61
BMI, kg/m²	22.1 ± 2.13	22.8 ± 2.45	21.4 ± 1.61
Systolic blood pressure, mmHg	124 ± 15	127 ± 15	121 ± 14
Diastolic blood pressure, mmHg	75 ± 9	78 ± 10	72 ± 7
Heart rate, bpm	66 ± 11	63 ± 11	69 ± 11
Glucose, g/L	0.91 ± 0.079	0.96 ± 0.070	0.87 ± 0.063
Creatinine, μmol/L	67.0 ± 11	75.1 ± 9.62	60.0 ± 6.24
Urea, mmol/L	4.02 ± 1.04	4.19 ± 0.974	3.86 ± 1.10
SGPT, μkat/L	0.34 ± 0.244	0.47 ± 0.236	0.23 ± 0.193
SGOT, μkat/L	0.35 ± 0.119	0.40 ± 0.113	0.31 ± 0.109
γ-GT, μkat/L	0.35 ± 0.333	0.52 ± 0.425	0.20 ± 0.087
Leukocytes, 10^9^/L	6.34 ± 1.41	6.51 ± 1.74	6.19 ± 1.09
Erythrocytes, 10^12^/L	4.59 ± 0.382	4.80 ± 0.408	4.41 ± 0.253
Hemoglobin, g/dL	14.4 ± 1.05	15.2 ± 0.91	13.7 ± 0.57
Hematocrit, %	42.6 ± 3.31	45.0 ± 2.71	40.4 ± 2.05
Platelets, 10^9^/L	258 ± 66.7	236 ± 49.1	278 ± 75.3
Lymphocytes, 10^9^/L	2.05 ± 0.437	2.06 ± 0.501	2.04 ± 0.389
Monocytes, 10^9^/L	0.54 ± 0.196	0.58 ± 0.215	0.49 ± 0.174
Basophils, 10^9^/L	0.072 ± 0.026	0.068 ± 0.0236	0.075 ± 0.0283
Polynuclear neutrophils, 10^9^/L	3.47 ± 1.26	3.54 ± 1.52	3.41 ± 1.03
Mean corpuscular volume, fL	92.8 ± 3.94	94.2 ± 4.36	91.7 ± 3.22
MCHC, g/dL	33.8 ± 0.65	33.7 ± 0.71	33.9 ± 0.60
Erythrocyte distribution width, %	13.7 ± 1.23	13.7 ± 0.77	13.6 ± 1.55
Mean platelet volume, fL	9.48 ± 1.04	9.56 ± 1.03	9.41 ± 1.08

1 Values are means ± SD. MCHC, mean corpuscular hemoglobin concentration; SGOT, serum glutamic-oxaloacetic transaminase; SGPT, serum glutamic pyruvic transaminase; γ-GT, gamma-glutamyltransferase.

**FIGURE 4:**
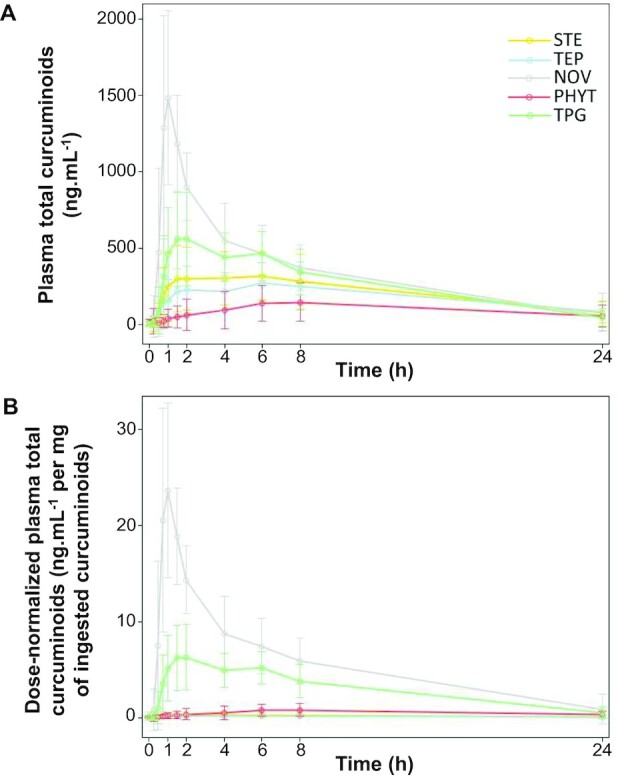
Twenty-four hour kinetics of plasma total curcuminoids after consumption of a single dose of the turmeric formulations STE, TEP, PHYT, NOV, and TPG by healthy human participants. (A) Concentrations of total curcuminoids; and (B) dose-normalized concentrations of total curcuminoids determined using UHPLC-tandem MS. Observed means ± SD on the ITT population, *n* = 30 for each formulation. Total curcuminoids = curcumin + curcumin sulfate + curcumin glucuronide + DMC + DMC sulfate + DMC glucuronide + BDMC + BDMC glucuronide + BDMC sulfate + THC + THC sulfate + THC glucuronide + HHC + HHC glucuronide + HHC sulfate. BDMC, bisdemethoxycurcumin; DMC, desmethoxycurcumin; HHC, hexahydrocurcumin; ITT, intent-to-treat; NOV, liquid micellar formulation; PHYT, phytosome formulation; STE, standard turmeric extract; TEP, piperine-curcuminoids combination; THC, tetrahydrocurcumin; TPG, Turmipure Gold formulation; UHPLC, ultra-high-pressure liquid chromatography.

**FIGURE 5:**
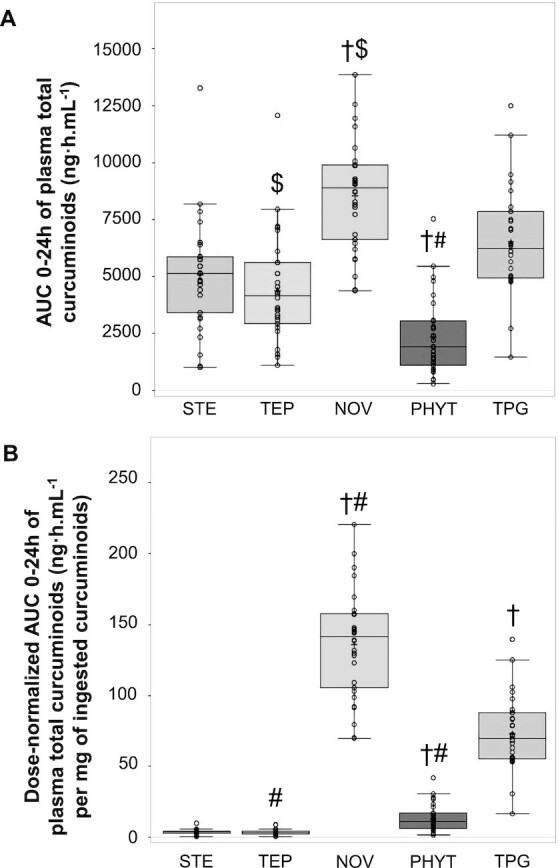
AUC 0–24h of plasma total curcuminoids after consumption of a single dose of the turmeric formulations STE, TEP, PHYT, NOV, and TPG by healthy human participants. (A) AUC 0–24h of total curcuminoids; and (B) dose-normalized AUC 0–24h of total curcuminoids determined using UHPLC-tandem MS. Boxplot representing the group medians, 25th and 75th percentiles, minima, and maxima of observed means on the ITT population, *n* = 30 for each formulation. †Significantly different from the reference STE product, *P* < 0.0001. $,#Significantly different compared with the TPG product: $*P* < 0.05, #*P* < 0.0001. Total curcuminoids = curcumin + curcumin sulfate + curcumin glucuronide + DMC + DMC sulfate + DMC glucuronide + BDMC + BDMC glucuronide + BDMC sulfate + THC + THC sulfate + THC glucuronide + HHC + HHC glucuronide + HHC sulfate. BDMC, bisdemethoxycurcumin; DMC, desmethoxycurcumin; HHC, hexahydrocurcumin; ITT, intent-to-treat; NOV, liquid micellar formulation; PHYT, phytosome formulation; STE, standard turmeric extract; TEP, piperine-curcuminoids combination; THC, tetrahydrocurcumin; TPG, Turmipure Gold formulation; UHPLC, ultra-high-pressure liquid chromatography.

All visits for all participants (*n* = 30) and 1648/1650 plasma time points were included in the analyses on the ITT population (see Supplemental Methods for kinetics missing data handling). The concentrations of total curcuminoids (curcumin, BMC, DBMC, and their metabolites) in plasma over time after oral administration of each of the 5 curcuminoid formulations were plotted (**Figure 4**A) and also expressed using a box plot to show variability in the study population (**Figure 5**A). No significant visit effect was identified for AUC 0–24h of total curcuminoids (*P* = 0.23) nor for dose-normalized AUC 0–24h of total curcuminoids (*P* = 0.23). Consequently, analyses were performed on all visits. However, some statistically significant visit effects were identified for individual curcuminoids or metabolites or secondary outcomes and are listed in **Supplemental Table 5**; therefore, for those parameters, data analysis was performed on visit 1 only.

### The proportions of individual metabolites after absorption

The methods developed for this study allowed the quantification of 15 individual curcuminoids, as well as various groups of these curcuminoids regarding their biochemical properties (Figure 1). Therefore, the proportions of individual metabolites and sum of the major groups expressed in percentages were calculated as the ratio of the AUC 0–24h of the compound or group of compounds of interest divided by the AUC 0–24h of total curcuminoids for the 5 formulations (**Table 3**). Plasma concentrations of unconjugated and conjugated curcuminoids indicate high interindividual variability. Interestingly, unconjugated curcumin and parent curcuminoids were minor, representing only 1.1% and 1.3%, respectively, of all metabolites quantified in plasma for the standard extract formulation STE. These proportions did not vary significantly for the other formulations.

**TABLE 3:** Contribution of the different curcuminoid(s) to the total metabolites after consumption of a single dose of the turmeric formulations STE, TEP, PHYT, NOV, and TPG by healthy human participants^1^

Considered curcuminoids (no. of metabolites), %	STE	TEP	PHYT	NOV	TPG
Curcumin (1)	1.1	1.2	1.6	0.3	0.9
DMC (1)	0.1	0.6	0.3	0.0	0.1
BDMC (1)	0.0	0.0	0.0	0.0	0.0
Curcumin + DMC + BDMC (3)	1.3	1.8	1.9	0.3	1.1
Curcumin glucuronide (1)	4.6	4.3	6.1	5.7	3.5
DMC glucuronide (1)	0.8	0.8	2.7	1.0	0.4
BDMC glucuronide (1)	0.1	0.1	0.3	0.1	0.0
Curcumin sulfate (1)	12.2	10.8	12.5	7.1	8.8
DMC sulfate (1)	2.3	2.5	5.2	0.8	1.5
BDMC sulfate (1)	0.8	0.8	0.8	1.2	0.1
Sum of parent compounds and their relative glucuronide and sulfate metabolites (9)^2^	22.1	21.1	29.4	16.2	15.5
THC (1)	0.0	0.0	0.0	0.0	0.0
THC glucuronide (1)	26.0	28.1	27.0	35.8	31.2
THC sulfate (1)	6.3	6.0	7.0	3.5	3.2
HHC (1)	0.0	0.0	0.0	0.0	0.0
HHC glucuronide (1)	18.6	16.8	13.9	21.0	22.2
HHC sulfate (1)	27.0	28.0	22.7	23.6	28.0
Curcumin and all its metabolites (9)^3^	95.8	95.3	90.9	96.9	97.8
Total curcuminoids (15)^4^	100	100	100	100	100

1 All proportions (expressed as percentage of total curcuminoids) were calculated as the ratio of the AUC 0–24h of the compound or group of compounds of interest divided by the AUC 0–24h of total curcuminoids × 100. BDMC, bisdemethoxycurcumin; DMC, demethoxycurcumin; HHC, hexahydrocurcumin; NOV, liquid micellar formulation; PHYT, phytosome formulation; STE, standard turmeric extract; TEP, piperine-curcuminoids combination; THC, tetrahydrocurcumin; TPG, Turmipure Gold formulation.

2 Sum of curcuminoids and their relative glucuronide and sulfate metabolites = curcumin + curcumin sulfate + curcumin glucuronide + DMC + DMC sulfate + DMC glucuronide + BDMC + BDMC glucuronide + BDMC sulfate.

3 Curcumin and all its metabolites = curcumin + curcumin sulfate + curcumin glucuronide + THC + THC sulfate + THC glucuronide + HHC + HHC glucuronide + HHC sulfate.

4 Total curcuminoids = curcumin + curcumin sulfate + curcumin glucuronide + DMC + DMC sulfate + DMC glucuronide + BDMC + BDMC glucuronide + BDMC sulfate + THC + THC sulfate + THC glucuronide + HHC + HHC glucuronide + HHC sulfate.

The sum of all parent curcuminoids including their glucuronide and sulfate metabolites ranged from 15.5% to 29.4% of the total metabolites, with greater proportions due to curcumin and its conjugated forms, with DMC and BDMC metabolites being less abundant (Table 3). Due to undetectable concentrations for most participants and formulations, the model could not estimate any pharmacokinetic parameters for the reduced forms of curcumin, THC and HHC (phase I metabolites). On the other hand, THC glucuronide and THC sulfate represented a high proportion of the total metabolites, varying respectively from 26% to 35.8% and 3.2% to 7%. Unexpectedly, and for all tested formulations, the plasma concentrations of the glucuronic acid and sulfate phase II conjugates of curcumin and all other curcuminoids were considerably higher than those of the unconjugated forms, with the sulfate conjugates usually predominating over the glucuronides. These results highlight the diversity of systemic curcuminoids after consumption of turmeric formulations (Table 3).

### Pharmacokinetics of unconjugated curcuminoids

Although unconjugated curcumin, DMC, and BDMC are commonly used to compare turmeric preparations, the 5 tested formulations did not differ in AUC 0–24h or Cmax (**Table 4**). Unlike curcumin, the unconjugated forms of DMC and BDMC were often near or below the limit of quantification (Table 4, Supplemental Table 4), explaining the similarities between the proportions of unconjugated curcumin and parent curcuminoids discussed above (Table 3). Lastly, reduced forms of curcumin THC and HHC (phase I metabolites), were undetectable for most formulations (data not shown).

**TABLE 4:** Pharmacokinetic parameters and relative bioavailabilities of unconjugated parent curcuminoids after consumption of a single dose of the turmeric formulations STE, TEP, PHYT, NOV, and TPG by healthy human participants^1^

	Curcumin					DMC					BDMC				
	STE	TEP	PHYT	NOV	TPG	STE	TEP	PHYT	NOV	TPG	STE	TEP	PHYT	NOV	TPG
AUC 0–8h, ng·h/mL	30.6 ± 75.3	16.4 ± 38.0	30.7 ± 68.7	12.1 ± 43.1	33.6 ± 83.8	5.0 ± 14.2	20.5 ± 104	5.2 ± 12.3	1.8 ± 7.50	5.7 ± 15.3	0.6 ± 1.96	0.2 ± 0.65	0.2 ± 0.56	0.1 ± 0.27	0.6 ± 1.47
AUC 0–24h, ng·h/mL	55.8 ± 106	54.1 ± 83.5	37.6 ± 70.9	25.2 ± 61.2	59.6 ± 118	7.4 ± 17.0	25.7 ± 107	6.4 ± 11.4	2.6 ± 8.36	9.4 ± 20.8	1.3 ± 4.06	0.6 ± 2.08	0.2 ± 0.55	0.1 ± 0.27	0.6 ± 1.47
Cmax, ng/mL	18.0 ± 37.0	12.9 ± 26.3	11.3 ± 22.1	9.1 ± 32.5	15.4 ± 36.5	3.2 ± 7.44	10.3 ± 49.0	2.2 ± 4.15	1.3 ± 5.44	2.7 ± 6.71	0.7 ± 1.43	0.2 ± 0.62	0.2 ± 0.41	0.2 ± 0.69	0.4 ± 0.87
AUC 0–8h normalized, ng·h/mL/mg	0.0 ± 0.07	0.0 ± 0.03	0.2 ± 0.47	0.2 ± 0.86	0.5 ± 1.13	0.0 ± 0.07	0.1 ± 0.45	0.2 ± 0.43	0.2 ± 0.68	0.4 ± 1.10	0.0 ± 0.10	0.0 ± 0.02^$^	0.1 ± 0.14	0.0 ± 0.18^$^	0.4 ± 1.14
AUC 0–24h normalized, ng·h/mL/mg	0.0 ± 0.09	0.0 ± 0.07^$^	0.3 ± 0.48	0.5 ± 1.22	0.8 ± 1.59*	0.0 ± 0.08	0.1 ± 0.47^$^	0.2 ± 0.40	0.2 ± 0.76	0.7 ± 1.50*	0.1 ± 0.21	0.0 ± 0.07^$^	0.0 ± 0.14	0.0 ± 0.18	0.4 ± 1.14
Cmax normalized, ng/mL/mg	0.0 ± 0.03	0.0 ± 0.02	0.1 ± 0.15	0.2 ± 0.65	0.2 ± 0.49	0.0 ± 0.03	0.0 ± 0.21	0.1 ± 0.14	0.1 ± 0.50	0.2 ± 0.48	0.0 ± 0.07	0.0 ± 0.02^$^	0.0 ± 0.11	0.1 ± 0.45	0.3 ± 0.67
Tmax, min	211 ± 430	362 ± 522	224 ± 367	132 ± 278	266 ± 417	60.0 ± 262	175 ± 377	233 ± 441	55.7 ± 264	94.1 ± 274	18.5 ± 49.0	64.0 ± 268	17.1 ± 70.0	2.0 ± 8.57	25.0 ± 66.0
Half-life, min	816 ± 1590	3520 ± 5410	1370 ± 2050	612 ± 816	750 ± 1140	64.0 ± 171	86.6 ± 311^nk^	69.0 ± 285^nk^	102 ± 485^nk^	1010 ± 4520^nk^	214 ± 1050	3.7 ± 18.7^nk^	17.9 ± 91.1^nk^	0.5 ± 2.40^nk^	5.9 ± 30.1^nk^
Terminal elimination rate constant, ng/h	0.1 ± 0.12	0.1 ± 0.31	0.3 ± 1.15	0.5 ± 1.53	0.1 ± 0.15	0.0 ± 0.12	0.3 ± 1.31^nk^	0.0 ± 0.01^nk^	0.0 ± 0.03^nk^	0.0 ± 0.09^nk^	0.2 ± 0.57	0.0 ± 0.09^nk^	0.0 ± 0.02^nk^	0.1 ± 0.62^nk^	0.0 ± 0.05^nk^
Relative bioavailability 0–8h	1.0 ± 0.00	42.7 ± 180^$^	143 ± 400	88.0 ± 237	342 ± 1270	1.0 ± 0.00	6.2 ± 18.0^nk^	28.5 ± 54.4^nk^	0.7 ± 2.11^nk^	55.6 ± 164	1.0 ± 0.00	0.0 ± 0.00^nk^	1.5 ± 4.34^nk^	0.0 ± 0.00^nk^	0.0 ± 0.00
Relative bioavailability 0–24h	1.0 ± 0.00	16.2 ± 41.0	58.0 ± 193	141 ± 435	156 ± 407	1.0 ± 0.00	7.6 ± 18.0^nk^	17.6 ± 45.8^nk^	0.7 ± 2.11^nk^	61.8 ± 163	1.0 ± 0.00	0.0 ± 0.00^nk^	1.5 ± 4.34^nk^	0.0 ± 0.00^nk^	0.0 ± 0.00^nk^

1 Values are means ± SD, *n* = 30. Size of data sets are available in Supplemental Table 6. *Significantly different compared with the reference STE product, *P* < 0.05; ^$^significantly different compared with the TPG product, *P* < 0.05. nk means the model was not estimated due to a high number of missing data or data not different from 0. BDMC, bisdemethoxycurcumin; Cmax, peak plasma concentration; DMC, demethoxycurcumin; NOV, liquid micellar formulation; PHYT, phytosome formulation; STE, standard turmeric extract; TEP, piperine-curcuminoids combination; Tmax, time to maximum concentration; TPG, Turmipure Gold formulation.

The AUC 0–24h values of unconjugated curcumin ranged from 25.2 to 59.6 ng·h/mL, and were brought respectively by the micellar preparation NOV and the colloidal suspension TPG. Cmax values ranged from 9.1 to 18.0 ng/mL, corresponding respectively to the micellar preparation NOV and the standard turmeric extract STE (Table 4). Interestingly, comparisons observed after dose normalization demonstrated an improved unconjugated curcumin absorption rate for the colloidal suspension TPG relative to other formulations, reaching significance when compared with the standard extract STE and the piperine-curcuminoid combination TEP (AUC 0–24h, *P* < 0.05 compared with both; Table 4).

### Pharmacokinetics of curcumin metabolites

This group of curcuminoids includes curcumin, its reduced forms THC and HHC, and all their respective glucuronic acid and sulfate conjugates (phase II metabolites). In fact, these 9 compounds together represented ∼90% of plasma curcuminoids after turmeric formulation consumption (Table 3). When looking at conjugate plasma concentrations in the tested formulations for curcumin, THC, and HHC, both glucuronide and sulfate forms demonstrated significant differences with either the standard turmeric extract STE or the new colloidal suspension TPG (**Tables 5** and **6**).

**TABLE 5:** Pharmacokinetic parameters and relative bioavailabilities of glucuronide-conjugated curcumin metabolites (curcumin, THC, and HHC) after consumption of a single dose of the turmeric formulations STE, TEP, PHYT, NOV, and TPG by healthy human participants^1^

	Curcumin glucuronide					THC glucuronide					HHC glucuronide				
	STE	TEP	PHYT	NOV	TPG	STE	TEP	PHYT	NOV	TPG	STE	TEP	PHYT	NOV	TPG
AUC 0–8h, ng·h/mL	79.7 ± 77.6	63.1 ± 40.0	45.0 ± 63.9^†,#^	338 ± 142^†,#^	98.2 ± 70.6	597 ± 395	455 ± 348^$^	155 ± 223^†,#^	1950 ± 561*	1140 ± 489	597 ± 395	455 ± 348^$^	155 ± 223^†,#^	1950 ± 561*	1140 ± 489
AUC 0–24h, ng·h/mL	236 ± 170	187 ± 96.7	142 ± 157^*,$^	491 ± 214^†,#^	226 ± 141	1320 ± 986	1230 ± 1110^#^	628 ± 973^†,$^	3050 ± 1150*	2030 ± 1090	946 ± 601	736 ± 536^#^	323 ± 291^†,#^	1790 ± 906^†^	1450 ± 765*
Cmax, ng/mL	33.9 ± 49.7	26.5 ± 44.7	25.6 ± 63.0^*,$^	295 ± 137 ^†,#^	42.5 ± 64.7	163 ± 70.6	154 ± 84.5^$^	72.7 ± 89.5^†,#^	711 ± 249^†^	277 ± 128	97.9 ± 66.6	70.0 ± 38.2^#^	31.7 ± 22.6^†,#^	330 ± 137^†,#^	165 ± 87.3*
AUC 0–8h normalized, ng·h/mL/mg	0.1 ± 0.07	0.1 ± 0.04^#^	0.3 ± 0.44^*,#^	6.7 ± 2.84^†,#^	1.3 ± 0.95^†^	0.5 ± 0.35	0.4 ± 0.31^#^	1.1 ± 1.52^#^	38.9 ± 11.2^†,#^	15.3 ± 6.59^†^	0.5 ± 0.35	0.4 ± 0.31^#^	1.1 ± 1.52^#^	38.9 ± 11.2^†,#^	15.3 ± 6.59^†^
AUC 0–24h normalized, ng·h/mL/mg	0.2 ± 0.15	0.2 ± 0.09^#^	1.0 ± 1.07^†,#^	9.8 ± 4.26^†,#^	3.0 ± 1.89^†^	1.2 ± 0.86	1.1 ± 0.99^#^	4.3 ± 6.65^#^	60.8 ± 23.0^†,#^	27.4 ± 14.6^†^	0.8 ± 0.53	0.7 ± 0.48^#^	2.2 ± 1.99^†,#^	35.7 ± 18.1^†,$^	19.5 ± 10.3^†^
Cmax normalized, ng/mL/mg	0.0 ± 0.04	0.0 ± 0.04^#^	0.2 ± 0.43^†,#^	5.9 ± 2.74^†,#^	0.6 ± 0.87^†^	0.1 ± 0.06	0.1 ± 0.08^#^	0.5 ± 0.61^*,#^	14.2 ± 4.97^†,#^	3.7 ± 1.72^†^	0.1 ± 0.06	0.1 ± 0.03^#^	0.2 ± 0.15^†,#^	6.6 ± 2.74^†,#^	2.2 ± 1.18^†^
Tmax, min	277 ± 285	289 ± 281	548 ± 497	51.5 ± 15.6^*,$^	177 ± 266	256 ± 278	322 ± 356	181 ± 207^*,$^	62.5 ± 18.9	161 ± 142	249 ± 153	293 ± 269	391 ± 246^*,#^	72.5 ± 57.0^†,#^	203 ± 160
Half-life,^2^ min	2100 ± 1970	3040 ± 6930	2230 ± 1860	706 ± 465*	1260 ± 1750*	1570 ± 3570	730 ± 1190	134 ± 330^†,#^	491 ± 628	1580 ± 2730	1570 ± 3570	730 ± 1190	134 ± 330^†,#^	491 ± 628	1580 ± 2730
Terminal elimination rate constant,^2^ ng/h	0.0 ± 0.02	0.0 ± 0.04	0.0 ± 0.02	0.1 ± 0.05*	0.1 ± 0.06*	0.1 ± 0.16	0.2 ± 0.30	0.0 ± 0.02	0.2 ± 0.11	0.1 ± 0.10	0.1 ± 0.16	0.2 ± 0.30	0.0 ± 0.02	0.2 ± 0.11	0.1 ± 0.10
Relative bioavailability 0–8h^2^	1.0 ± 0.00	1.2 ± 1.19 ^#^	7.0 ± 12.0^#^	143 ± 75.0^#^	29.5 ± 26.1	1.0 ± 0.00	0.9 ± 1.16^#^	2.1 ± 2.94^#^	102 ± 104^#^	39.8 ± 45.4	1.0 ± 0.00	0.9 ± 1.16^#^	2.1 ± 2.94^#^	102 ± 104^#^	39.8 ± 45.4
Relative bioavailability 0–24h^2^	1.0 ± 0.00	1.1 ± 0.84^#^	7.7 ± 15.5^#^	63.2 ± 34.3^#^	19.9 ± 13.4	1.0 ± 0.00	1.8 ± 4.34^#^	4.6 ± 8.90^#^	90.8 ± 113^$^	32.5 ± 22.3	1.0 ± 0.00	1.2 ± 1.14^#^	3.5 ± 3.56^#^	93.2 ± 159^$^	34.8 ± 26.7

1 Values are means ± SD, *n* = 30. Size of data sets are available in Supplemental Table 6. ^*,†^Statistically significant compared with the reference STE product: *P < 0.05, ^†^*P* < 0.0001. ^$,#^Statistically significant compared with the TPG product: ^$^*P* < 0.05, ^#^*P* < 0.0001. Cmax, peak plasma concentration; HHC, hexahydrocurcumin; NOV, liquid micellar formulation; PHYT, phytosome formulation; STE, standard turmeric extract; TEP, piperine-curcuminoids combination; THC, tetrahydrocurcumin; Tmax, time to maximum concentration; TPG, Turmipure Gold formulation.

2 For these parameters, *n* = 9–28.

**TABLE 6:** Pharmacokinetic parameters and relative bioavailabilities of sulfate-conjugated curcumin metabolites (curcumin, THC, and HHC) after consumption of a single dose of the turmeric formulations STE, TEP, PHYT, NOV, and TPG by healthy human participants^1^

	Curcumin sulfate					THC sulfate					HHC sulfate				
	STE	TEP	PHYT	NOV	TPG	STE	TEP	PHYT	NOV	TPG	STE	TEP	PHYT	NOV	TPG
AUC 0–8h, ng·h/mL	250 ± 192	180 ± 117	115 ± 177	365 ± 171	285 ± 226	100 ± 172	99.2 ± 149	30.8 ± 108^*,$^	122 ± 188	86.9 ± 134	100 ± 172	99.2 ± 149	30.8 ± 108^*,$^	122 ± 188	86.9 ± 134
AUC 0–24h, ng·h/mL	618 ± 290	474 ± 208	291 ± 170	609 ± 314	575 ± 320	317 ± 603	264 ± 442	164 ± 486	296 ± 528	211 ± 489	1370 ± 1150	1230 ± 810^#^	528 ± 256^†,#^	2010 ± 859^†^	1830 ± 744*
Cmax, ng/mL	59.6 ± 54.2	42.3 ± 35.4	27.1 ± 34	125 ± 59.7^*,$^	63.3 ± 57.6	59.5 ± 74.8	74.2 ± 83.7	24.4 ± 55.8^*,$^	62.7 ± 79.3	61.1 ± 75.5	128 ± 100	98.8 ± 54.9^#^	47.6 ± 27.3^†,#^	344 ± 163^†,#^	197 ± 101^†^
AUC 0–8h normalized, ng·h/mL/mg	0.2 ± 0.17	0.2 ± 0.10^#^	0.8 ± 1.21^†,#^	7.3 ± 3.40^†,$^	3.8 ± 3.05^†^	0.1 ± 0.15	0.1 ± 0.13	0.2 ± 0.74	2.4 ± 3.74	1.2 ± 1.81	0.1 ± 0.15	0.1 ± 0.13	0.2 ± 0.74	2.4 ± 3.74	1.2 ± 1.81
AUC 0–24h normalized, ng·h/mL/mg	0.5 ± 0.25	0.4 ± 0.19^#^	2.0 ± 1.16^†,$^	12.1 ± 6.25^†^	7.7 ± 4.30^†^	0.3 ± 0.53	0.2 ± 0.39	1.1 ± 3.32	5.9 ± 10.5	2.8 ± 6.59	1.2 ± 1.01	1.1 ± 0.72^#^	3.6 ± 1.75^†,#^	40.1 ± 17.1^†,#^	24.6 ± 10.0^†^
Cmax normalized, ng/mL/mg	0.1 ± 0.05	0.0 ± 0.03^#^	0.2 ± 0.23^†,$^	2.5 ± 1.19^†,#^	0.9 ± 0.78^†^	0.1 ± 0.07	0.1 ± 0.07	0.2 ± 0.38	1.2 ± 1.58	0.8 ± 1.02	0.1 ± 0.09	0.1 ± 0.05^#^	0.3 ± 0.19^†,#^	6.9 ± 3.24^†,#^	2.7 ± 1.36^†^
Tmax, min	347 ± 137	375 ± 101^$^	438 ± 220^$^	73.0 ± 39.6^†,#^	286 ± 127	97.0 ± 140	189 ± 361	114 ± 300	174 ± 181	176 ± 279	232 ± 266	200 ± 145	490 ± 407^†,#^	88.5 ± 57.4^†,$^	176 ± 112
Half-life, min	973 ± 987	680 ± 468	1110 ± 1670	602 ± 1340	1420 ± 5340	272 ± 903	98.0 ± 259	0.0 ± 0.00	184 ± 400	195 ± 575	272 ± 903	98.0 ± 259	0.0 ± 0.00	184 ± 400	195 ± 575
Terminal elimination rate constant, ng/h	0.1 ± 0.06	0.1 ± 0.04	0.1 ± 0.08	0.2 ± 0.09	0.1 ± 0.11	0.2 ± 0.38	0.1 ± 0.22	0.0 ± 0.00^$^	0.1 ± 0.17	0.3 ± 0.47	0.2 ± 0.38	0.1 ± 0.22	0.0 ± 0.00^$^	0.1 ± 0.17	0.3 ± 0.47
Relative bioavailability 0–8h	1.0 ± 0.00	1.1 ± 1.08^#^	6.1 ± 12.6^#^	49.2 ± 39.1^$^	25.0 ± 24.6	1.0 ± 0.00	1.0 ± 1.17^$^	3.4 ± 9.08^$^	21.2 ± 30.6	15.9 ± 20.3	1.0 ± 0.00	1.0 ± 1.17^$^	3.4 ± 9.08^$^	21.2 ± 30.6	15.9 ± 20.3
Relative bioavailability 0–24h	1.0 ± 0.00	0.9 ± 0.60^#^	4.7 ± 4.38^$^	28.7 ± 30.3	16.5 ± 9.86	1.0 ± 0.00	1.6 ± 2.84	13.1 ± 38.3	74.6 ± 125	26.7 ± 48.3	1.0 ± 0.00	1.0 ± 0.57^#^	3.7 ± 1.91^#^	45.3 ± 27.4	25.8 ± 12.7

1 Values are means ± SD, *n* = 30. Size of data sets are available in Supplemental Table 6. ^*,†^Statistically significant compared with the reference STE product: *P < 0.05, ^†^*P* < 0.0001. ^$,#^Statistically significant compared with the TPG product: ^$^*P* < 0.05, ^#^*P* < 0.0001. Cmax, peak plasma concentration; HHC, hexahydrocurcumin; NOV, liquid micellar formulation; PHYT, phytosome formulation; STE, standard turmeric extract; TEP, piperine-curcuminoids combination; THC, tetrahydrocurcumin; Tmax, time to maximum concentration; TPG, Turmipure Gold formulation.

The plasma glucuronides of curcumin, THC, and HHC varied respectively from 3.5% to 6.1%, 26% to 35.8%, and 13.9% to 22.2% between all tested formulations (Table 3). When using the standard turmeric extract STE as a reference, the micellar preparation NOV led to significantly higher glucuronide AUC 0–24h and Cmax for curcumin, THC, and HHC (all *P* < 0.05). In contrast, the phytosome formulation PHYT glucuronide AUC 0–24h and Cmax were significantly lower for curcumin, THC, and HHC (all *P* < 0.05). Despite similar levels for curcumin glucuronide and THC glucuronide with the standard turmeric extract STE, the new colloidal suspension TPG produced significantly higher glucuronide levels for HHC (AUC 0–24h and Cmax, *P* < 0.05). The colloidal suspension TPG also showed significantly higher glucuronide AUC 0–24h and Cmax than did the phytosome formulation PHYT for curcumin, THC, and HHC (all *P* < 0.05). Significantly higher AUC 0–24h and Cmax were also observed when compared with the piperine-curcuminoid combination TEP for both THC and HHC glucuronides (all *P* < 0.05, Table 5).

The plasma sulfate conjugate proportions of total curcuminoids quantified in all tested formulations represented 7.1% to 12.5%, 3.2% to 7%, and 22.7% to 28%, respectively, for curcumin, THC, and HHC (Table 3). The comparisons with the standard turmeric extract STE showed significantly higher sulfate conjugate levels of curcumin (only Cmax, *P* < 0.05) and HHC (AUC 0–24h and Cmax, *P* < 0.0001) for the micellar preparation NOV. Inversely, the phytosome formulation PHYT produced significantly lower levels than did the standard turmeric extract STE for the sulfate forms of THC (only Cmax, *P* < 0.05) and HHC (AUC 0–24h and Cmax, *P* < 0.0001). Similar to the glucuronide forms, the novel colloidal suspension TPG produced significantly higher sulfate levels for HHC (AUC 0–24h and Cmax, *P* < 0.05) than the standard turmeric extract STE. The colloidal suspension TPG also showed significantly higher sulfate levels than the phytosome formulation PHYT for THC (Cmax, *P* < 0.05) and HHC (AUC 0–24h and Cmax, *P* < 0.0001), as well as significantly higher levels when compared with the piperine-curcuminoid combination (TEP) for HHC sulfate (AUC 0–24h and Cmax, *P* < 0.0001; Table 6).

In contrast to studying only unconjugated curcuminoids, this study of 9 curcumin metabolites individually and as a group, allowed the clear distinction between all the turmeric formulations tested over 24 h after consumption. Indeed, when compared with both the standard extract STE and the colloidal suspension TPG, significantly lower absorption levels were observed for the phytosome formulation PHYT (AUC 0–24h and Cmax, *P* < 0.0001), and higher absorption levels for the micellar preparation NOV (AUC 0–24h and Cmax compared with STE and Cmax only compared with TPG, all *P* < 0.0001). Importantly, in addition to higher absorption levels than the phytosome formulation PHYT, these results demonstrated superior AUC 0–24h and Cmax for the colloidal suspension TPG when compared with both the standard extract STE and the piperine-curcuminoid combination TEP (all *P* < 0.05; Table 7).

**TABLE 7:** Pharmacokinetic parameters and relative bioavailabilities of total curcuminoids, 3, 9, and 15 metabolites, after consumption of a single dose of the turmeric formulations STE, TEP, PHYT, NOV, and TPG by healthy human participants^1^

	Total parent curcuminoids^2^					Total curcumin metabolites^3^					Total curcuminoids^4^				
	STE	TEP	PHYT	NOV	TPG	STE	TEP	PHYT	NOV	TPG	STE	TEP	PHYT	NOV	TPG
Metabolites quantified, *n*	3					9					15				
AUC 0–8h, ng·h/mL	36.2 ± 89.9	37.1 ± 138	36.1 ± 81.4	13.9 ± 50.6	39.9 ± 99.8	2100 ± 1090	1620 ± 719^#^	654 ± 616^†,#^	5020 ± 1190^†,$^	3330 ± 1110*	2200 ± 1110	1730 ± 723 ^#^	738 ± 686^†,#^	5130 ± 1200^†,$^	3410 ± 1140*
AUC 0–24h, ng·h/mL	64.4 ± 123	80.3 ± 176	44.2 ± 81.7	27.8 ± 68.8	69.6 ± 139	4860 ± 2380	4170 ± 2330^$^	2110 ± 1660^†,#^	8280 ± 2340^†^	6370 ± 2250*	5080 ± 2410	4380 ± 2330^$^	2330 ± 1730^†,#^	8540 ± 2350^†,$^	6520 ± 2280
Cmax, ng/mL	21.5 ± 45.4	19.0 ± 51.1	13.4 ± 26.5	10.3 ± 37.4	18.3 ± 43.9	426 ± 188	356 ± 142^#^	190 ± 132^†,#^	1690 ± 436^†,#^	664 ± 269*	445 ± 193	373 ± 142^#^	209 ± 144^†,#^	1760 ± 454^†,#^	678 ± 273*
AUC 0–8h normalized, ng·h/mL/mg	0.0 ± 0.07	0.0 ± 0.10	0.2 ± 0.45	0.2 ± 0.81	0.4 ± 1.12	1.8 ± 0.95	1.4 ± 0.64^#^	4.5 ± 4.21^†,#^	99.9 ± 23.7^†,#^	44.9 ± 15.0^†^	1.6 ± 0.81	1.2 ± 0.52^#^	4.1 ± 3.83^†,#^	81.8 ± 19.2^†,#^	38.1 ± 12.7^†^
AUC 0–24h normalized, ng·h/mL/mg	0.0 ± 0.09	0.1 ± 0.13^$^	0.2 ± 0.46	0.4 ± 1.10	0.8 ± 1.55*	4.3 ± 2.08	3.7 ± 2.08^#^	14.4 ± 11.3^†,#^	165 ± 46.7^†,#^	85.8 ± 30.3^†^	3.7 ± 1.75	3.2 ± 1.69^#^	13.0 ± 9.65^†,#^	136 ± 37.4^†,#^	72.9 ± 25.5^†^
Cmax normalized, ng/mL/mg	0.0 ± 0.03	0.0 ± 0.04	0.1 ± 0.15	0.2 ± 0.60	0.2 ± 0.49	0.4 ± 0.16	0.3 ± 0.13^#^	1.3 ± 0.91^†,#^	33.6 ± 8.69^†,#^	8.9 ± 3.62^†^	0.3 ± 0.14	0.3 ± 0.10^#^	1.2 ± 0.80^†,#^	28.1 ± 7.23^†,#^	7.6 ± 3.06^†^
Tmax, min	163 ± 363	373 ± 518	286 ± 425	179 ± 366	264 ± 418	250 ± 175	324 ± 344	379 ± 248^*,$^	62.0 ± 17.9^†,#^	192 ± 143	257 ± 182	330 ± 341	375 ± 249^*,$^	61.0 ± 18.5^†,#^	190 ± 148
Half-life, min	1130 ± 2010	2190 ± 3320	1480 ± 2180	647 ± 863	815 ± 1130	904 ± 1780	561 ± 381^$^	719 ± 589^$^	312 ± 254*	317 ± 173*	788 ± 1230	505 ± 265^$^	640 ± 399^$^	337 ± 279*	318 ± 154*
Terminal elimination rate constant, ng/h	0.1 ± 0.12	0.1 ± 0.34	0.4 ± 1.33	0.5 ± 1.64	0.1 ± 0.08	0.1 ± 0.05	0.1 ± 0.04^$^	0.1 ± 0.04^$^	0.2 ± 0.05*	0.2 ± 0.06*	0.1 ± 0.05	0.1 ± 0.04^$^	0.1 ± 0.05^$^	0.2 ± 0.05*	0.2 ± 0.06*
Relative bioavailability 0–8h	1.0 ± 0.00	28.5 ± 91.3^$^	49.0 ± 95.5	88.0 ± 242	171 ± 471	1.0 ± 0.00	1.1 ± 0.98 ^#^	2.8 ± 2.36^#^	79.4 ± 69.3^#^	31.9 ± 21.5	1.0 ± 0.00	1.1 ± 1.07^#^	2.9 ± 2.50^#^	72.2 ± 58.7^#^	30.6 ± 20.0
Relative bioavailability 0–24h	1.0 0.00	28.8 95.4	72.3 229	141 ± 422	170 ± 449	1.0 ± 0.00	1.1 ± 0.81^#^	4.1 ± 4.12^#^	53.0 ± 42.3^#^	25.0 ± 16.3	1.0 ± 0.00	1.1 ± 0.79^#^	4.2 ± 4.15^#^	49.7 ± 37.8^#^	24.2 ± 15.5

1 Values are means ± SD, *n* = 30. Size of data sets are available in Supplemental Table 6. ^*,†^Statistically significant compared with the reference STE product: *P < 0.05, ^†^*P* < 0.0001. ^$,#^Statistically significant compared with the TPG product: ^$^*P* < 0.05, ^#^*P* < 0.0001. BDMC, bisdemethoxycurcumin; Cmax, peak plasma concentration; DMC, demethoxycurcumin; HHC, hexahydrocurcumin; NOV, liquid micellar formulation; PHYT, phytosome formulation; STE, standard turmeric extract; TEP, piperine-curcuminoids combination; THC, tetrahydrocurcumin; Tmax, time to maximum concentration; TPG, Turmipure Gold formulation.

^2^Total parent curcuminoids (unconjugated) = curcumin + DMC + BDMC.

^3^Curcumin and all its metabolites = curcumin + curcumin sulfate + curcumin glucuronide + THC + THC sulfate + THC glucuronide + HHC + HHC glucuronide + HHC sulfate.

^4^Total curcuminoids (parents + reduced and their conjugates) = curcumin + curcumin sulfate + curcumin glucuronide + DMC + DMC sulfate + DMC glucuronide + BDMC + BDMC glucuronide + BDMC sulfate + THC + THC sulfate + THC glucuronide + HHC + HHC glucuronide + HHC sulfate.

### Pharmacokinetics and bioavailability of total curcuminoids

This group of curcuminoids encompasses all the parents, reduced (phase I) and conjugated (phase II) compounds, which together represent 15 curcuminoids (curcumin, curcumin sulfate, curcumin glucuronide, DMC, DMC sulfate, DMC glucuronide, BDMC, BDMC glucuronide, BDMC sulfate, THC, THC sulfate, THC glucuronide, HHC, HHC glucuronide, and HHC sulfate). Plasma AUC 0–24h and Cmax ranges fluctuated respectively from 2330 to 8540 ng·h/mL and 209 to 1760 ng/mL (Figures 4A and 5A; Table 7), showing significant variations between the different formulations when compared with either the standard extract STE or the colloidal suspension TPG. Indeed, the micellar preparation NOV displayed significantly higher AUC 0–24h and Cmax than did the standard extract STE, whereas the phytosome formulation PHYT had significantly lower AUC 0–24h and Cmax (all *P* < 0.0001). Similar significant differences were observed with the colloidal suspension TPG when compared with the micellar preparation NOV (AUC 0–24h and Cmax, *P* < 0.05). However, the new colloidal suspension TPG brought significantly higher concentrations of plasma total curcuminoids than did the phytosome formulation PHYT, the piperine-curcuminoid combination TEP (AUC 0–24h and Cmax, all *P* < 0.05), or the standard extract STE (Cmax only, *P* < 0.05) (Table 7).

The primary study end point defined for this clinical trial was the dose-normalized AUC 0–24h of total plasma curcuminoids (expressed in ng·h/mL/mg), observable for all formulations in Figures 4B and 5A, and tabulated in Table 7. After dose normalization, plasma concentrations and corresponding AUC 0–24h of total curcuminoids were significantly lower for the standard extract STE than for the micellar preparation NOV, the phytosome formulation PHYT, or the colloidal suspension TPG (all *P* < 0.0001). Despite significantly higher AUC 0–24h and Cmax for the micellar preparation NOV (all *P* < 0.0001), it is interesting to highlight that the total curcuminoid concentrations observed after dose normalization confirmed greater pharmacokinetics AUC 0–24h and Cmax for the new colloidal suspension TPG than for the phytosome formulation PHYT or the piperine-curcuminoid combination TEP (all *P* < 0.0001). Importantly, the significantly higher total curcuminoids AUC 0–24h after dose normalization for the new colloidal suspension TPG, when compared with the standard extract STE, validated the primary hypothesis of this trial.

### Other pharmacokinetic parameters

In addition to AUC and Cmax, Tmax, *t*_1/2_, and λz were determined. In addition to producing higher AUC and Cmax for total curcuminoids, the micellar NOV and colloidal TPG formulations reduced Tmax values, notably for glucuronides of curcumin metabolites, sulfate conjugates of curcumin and HHC, and for total curcuminoids (Tables 5–7). After absorption, total curcuminoids showed shorter *t*_1/2_ values of 337 ± 279 and 318 ± 154 min for the micellar NOV and colloidal TPG formulations, respectively, compared with the standard extract STE (*t*_1/2_ = 788 ± 1230 min,  *P* < 0.05) (Table 7). Although the *t*_1/2_ of total curcuminoids was shorter for the micellar NOV and colloidal TPG formulations, systemic concentrations were significantly higher before (all *P* < 0.05), and after dose normalization (all *P* < 0.0001), especially considering smaller curcuminoid doses compared with the standard extract STE (respectively 95.4% and 93.5% lower; Tables 1 and 7).

The effect of sex was investigated for all of the pharmacokinetic parameters. No significant formulation–sex interaction was identified for AUC 0–24h of total curcuminoids or the individually quantified metabolites. A formulation–sex interaction was observed for Cmax of total curcuminoids (*P* < 0.05), and both Cmax and Tmax for total curcumin metabolites and HHC glucuronide (*P* < 0.05).

## Discussion

Unlike previous human pharmacokinetic studies of curcuminoids, this investigation is unique in that it compares multiple curcuminoid formulations available on the market at their daily recommended dosages. Comparing the 5 formulations at exactly the same curcuminoid dosage could have been scientifically meaningful. However, the approach described here is consistent and of interest for consumers, because curcuminoid bioavailability for each formulation has been assessed at dosages relevant to the consumer. Therefore, in addition to the specific ingredient contents, consumers will be able to make informed decisions regarding which turmeric formulation to choose based on their real curcuminoid absorption capacity. Another unique aspect of this investigation is that multiple pharmacokinetic parameters were measured for the 3 most abundant curcuminoids in turmeric (curcumin, BMC, and BDMC) as well as their major metabolites. A new method was specifically developed to allow quantification of numerous metabolites formed from these 3 curcuminoids and to understand better the curcuminoid bioavailability and metabolic transformation after oral administration of the different formulations.

The objective of this study was to assess the pharmacokinetics of a new natural turmeric dried colloidal suspension, TPG, to allow comparisons with the other turmeric formulations, NOV, TEP, PHYT, and a standard turmeric extract, STE, at their recommended dosages. The primary outcome of this trial validated our hypothesis that administration of 300 mg TPG would result in higher absorption levels of total curcuminoids after dose normalization, when compared with 1500 mg STE. In addition, comparisons of pharmacokinetics between each formulation allowed the estimation of curcuminoids and finished product quantities to be consumed to reach bioequivalence. Thus, the intakes for all formulations were calculated to provide equivalent AUC 0–24h to that of 300 mg of the colloidal suspension TPG (6520 ng · h/mL total curcuminoids), and resulted in 1920 mg, 2260 mg, 2870 mg, and 763 mg of STE, TEP, PHYT, and NOV, respectively (**Table 8**). Based on this extrapolation, the colloidal suspension TPG could demonstrate an enhanced bioavailability at a low dose. Finally, the relative bioavailability from 0 to 24 h of total curcuminoids in healthy volunteers for the colloidal suspension TPG (300 mg) was 24-fold higher than the standard turmeric extract STE (1500 mg). When compared with the other turmeric formulations, 300 mg of the new colloidal suspension was 22-fold more bioavailable than 1515 mg of the piperine-curcuminoid combination TEP, 6-fold more than 1000 mg of the phytosome formulation PHYT, and 2-time less than 1000 mg of the micellar preparation NOV (Table 7).

**TABLE 8:** Calculation of the quantity of curcuminoids (and finished products) to be ingested for each formula to reach the same AUC 0–24h as observed after consumption of 300 mg of TPG by healthy human participants (6520 ng·h/mL)^1^

	STE	TEP	PHYT	NOV	TPG
Dose-normalized AUC 0–24h of total curcuminoids, ng·h/mL/mg	3.7	3.2	13.0	136	72.9
Expected AUC 0–24h of total curcuminoids, ng·h/mL	6520				
Quantity of curcuminoids (finished product) to be consumed,^2^ mg	1760 (1920)	2040 (2260)	502 (2870)^3^	48 (763)^4^	90 (300)

1 NOV, liquid micellar formulation; PHYT, phytosome formulation; STE, standard turmeric extract; TEP, piperine-curcuminoids combination; TPG, Turmipure Gold formulation.

^2^The calculation is done using the corresponding dose normalized AUC 0–24h obtained for each product to reach 6520 ng·h/mL, and the quantity 0of product is therefore calculated based on the curcuminoids content quantified for each product (from Table 1).

3 Here we considered phytosome formulation (PHYT) dose-normalized AUC to be the same as that obtained at 1000 mg in our study despite the dose obtained from calculation being higher. However, because relative bioavailability is dose dependent (see Discussion), the dose equivalence may be inferior to 2870 mg because relative bioavailability would be higher.

4 Here we considered micellar formulation (NOV) dose-normalized AUC to be the same as that obtained at 1000 mg in our study despite the dose obtained from calculation being lower. However, because relative bioavailability is dose dependent (see Discussion), the dose equivalence may be superior to 763 mg because relative bioavailability would be lower.

Due to limited curcuminoid absorption, it is commonly observed that pharmacokinetic studies of turmeric formulations intended to demonstrate improved bioavailability have used either a substantial dose of the product or a very low dose of their comparative (e.g., STE). As an example, Schiborr et al. (34) reported bioavailability enhancement for the micellar preparation, though the dosages used were much higher than those recommended by the manufacturers. Participants were administered 7519 mg of the micellar preparation NOV and only 526 mg standard turmeric extract STE (containing 500 mg curcuminoids). In these experimental settings, it is not surprising to observe that relative plasma concentrations of curcumin were 185-fold higher for the micellar preparation NOV than for the standard extract STE. In our study, participants consumed the same product according to the manufacturer's recommendations, that is, 1000 mg containing 62.7 mg curcuminoids, as well as a recommended efficacy dosage of 1500 mg of standard extract STE. By using this lower dosage (1000 mg compared with 7519 mg), our results showed an improvement in the curcumin bioavailability of only 35.5-fold instead of 185-fold relative to the standard extract STE. Other studies have also shown that curcuminoid relative bioavailability varies with the given doses. Cuomo et al. (33) showed that the phytosome formulation provided 27-fold higher relative absorption of curcumin and its conjugates when administering 209 mg curcuminoids, increasing to 32-fold higher relative absorption at a dosage of 376 mg curcuminoids. In the present study, the phytosome formulation PHYT, given at a dosage containing 179 mg curcuminoids, increased bioavailability of curcumin and its conjugates 5.5-fold relative to the standard extract STE (data not shown). In another study, Kumar et al. (41) found that the relative bioavailability of unconjugated curcuminoids increased 46-fold and 25-fold when administering 391 mg and 97.7 mg of curcuminoids, respectively. In the present study, commercial formulations were compared as marketed and used by consumers, thus limiting bias in the bioavailability evaluation due to specific unusual and unlikely usable dosages.

Another important result of this investigation concerns the piperine-curcuminoid combination TEP, which has been reported to enhance absorption and increase systemic concentration of curcumin in rats and humans (42). Piperine is a well-known inhibitor of drug metabolism, including glucuronidation, and can alter the bioavailability of many drugs (32). However, our results showed that the piperine-curcuminoid combination TEP did not improve curcuminoid bioavailability with respect to unconjugated curcumin, the 3 parent curcuminoids, the 9 curcumin metabolites, or even the 15 total curcuminoids evaluated. The different outcomes published in the previous study by Shoba et al. (42) could be attributed to the smaller number of participants (8 instead of 30), the shorter kinetic duration (3 h compared with 24 h in the present study, in which the Tmax of unconjugated curcumin was 6 h), and the quantification of only 1 instead of 15 curcuminoids.

Based on the individual quantification of 15 curcuminoid metabolites, this study demonstrated that unconjugated curcumin, DMC, and BDMC represented only 1% of the total plasma curcuminoids following oral administration of a variety of turmeric formulations. Therefore, curcumin plasma concentration reached a maximum of 18–21.5 ng/mL in contrast to >400 ng/mL for all metabolites combined. Although glucuronic acid and sulfate conjugates of curcumin, DMC, and BDMC represented ∼20% of the total curcuminoids found in blood, THC glucuronide, THC sulfate, HHC glucuronide, and HHC sulfate were identified as the most abundant metabolites in human plasma. Interestingly, unconjugated THC and HHC were not detected (Table 3). Previous studies addressing the effects of various formulations on bioavailability only measured unconjugated curcumin, DMC, and BDMC. Besides ignoring the majority of curcuminoids, the former approach would tend to overestimate relative bioavailability. For example, Gota et al. (43) detected no significant unconjugated curcumin in human plasma following oral administration of the standard extract containing 95% curcuminoids and therefore could not calculate the corresponding AUC or relative bioavailability. Antony et al. (44) reported bioavailability enhancement of 6.93-fold with respect to unconjugated curcumin (although the difference in AUC values were not statistically tested) when comparing a curcuminoids–essential oil combination with a standard extract. A more recent study by Jäger et al. (45) included 53% of the metabolites evaluated in the present investigation and found the relative bioavailability of the same curcuminoids–essential oil combination enhanced only 1.3-fold. For comparison, we found that the relative bioavailability of the colloidal suspension TPG over 8 h is 342-fold higher than the standard turmeric extract STE when only unconjugated curcumin is considered (no statistical difference on AUC 0–8h and Cmax; Table 4). This value is reduced to 30.6-fold, but reached significance when total curcuminoids are considered (both AUC 0–8h and Cmax, *P* < 0.05; Table 7). Altogether, this extended set of data highlights the necessity to consider not only unconjugated curcumin, but also a wider range of metabolites to assess the bioavailability of curcuminoids.

The characterization of each curcuminoid proportional bioavailability, based on 5 different turmeric formulations, drew new perspectives regarding our understanding of their biological efficacy. To date, there is no consensus whether the activities of turmeric extract are due to unconjugated curcumin, DMC, or BDMC, or to their phase I and phase II metabolites. A review of in vitro studies comparing the antioxidant, anti-inflammatory, antimutagenic, antidiabetic, and anticarcinogenic activities of BDMC, DMC, and curcumin reported 11 references showing BDMC was less active, 11 indicating BDMC was more active, and 4 showing that BDMC, DMC, and curcumin were equivalent (23). This review also reported that 13 studies indicated that THC was more potent than curcumin, 1 study showed equal potency, and 8 studies showed lower potency. Among the few studies that have investigated the activities of curcumin conjugates, Ireson et al. (46) showed that curcumin sulfate was less effective than unconjugated curcumin in reducing phorbol ester–induced PGE_2_ production in human cells. Similarly, Pal et al. (47) demonstrated that curcumin monoglucuronide and diglucuronide displayed very little antiproliferative activity and had no effect on NF-κB. Even in the case of a lower activity of glucuronide compared with unconjugated curcuminoids, it has been demonstrated that they might become deconjugated in tissues, as shown for other phytochemicals like quercetin and hydroxytyrosol (48, 49). A recent study in mice bone cells by Kunihiro et al. (50) demonstrated that β-glucuronidase can hydrolyze curcumin monoglucuronide and release curcumin to act locally within bones. Ozawa et al. (51) demonstrated that an increase in blood curcumin glucuronide concentration causes an increase in unconjugated curcumin, resulting in the suppression of human colon carcinomas implanted in mice. Taken together, these in vitro data emphasize the fact that all metabolites from curcumin, DMC, and BDMC might contribute to the pharmacological activities of turmeric, and that future studies of turmeric bioavailability and efficacy should not be limited to unconjugated curcumin.

Whereas the molecular structures of curcumin and curcuminoids are responsible for their free radical scavenging activity, other antioxidant and anti-inflammatory properties of curcuminoids have been strongly associated with the regulation of numerous transcription factors, cytokines, protein kinases, adhesion molecules, and redox enzymes that have been linked to aging and pathological conditions. Subsequently, biological efficacy of various turmeric extracts and formulations have shown health benefits in both preclinical and clinical studies, and have been reviewed elsewhere (31, 52–57). As explained earlier, a major limitation of these extracts is their extremely low oral bioavailability, caused by low absorption, rapid metabolism, and rapid excretion following ingestion; often, higher doses or specific formulations are necessary to achieve significant pharmacological effects. This dedicated pharmacokinetic trial demonstrated that the new turmeric colloidal suspension TPG enhanced bioavailability such that 300 mg provided 1.3-fold more plasma curcuminoids than 1500 mg of the standard extract STE (the classical observed efficacy dose). Furthermore, 300 mg of TPG produced 1.5-fold higher curcuminoid plasma concentrations than 1515 mg of the piperine-curcuminoid combination TEP, 2.8-fold higher than 1000 mg of the phytosome formulation PHYT, and the same amount of total curcuminoids in blood as 763 mg of the micellar preparation NOV. The delivery efficacy of the product is therefore not linked only to the amount of curcuminoids contained in the formulation but rather its ability to improve the bioavailability of its components. The biological efficacy of the standard extract STE at 1500 mg had been demonstrated previously (24–31), and 300 mg of the colloidal suspension TPG showed at least equivalent bioavailability, suggesting that fewer capsules would be needed to produce comparable benefits.

The safety profiles of all products tested during this study showed no problems on a single-dose basis. Although turmeric extracts have a long history of use worldwide with a good safety profile and no particular concerns documented for higher dosages, no dedicated human clinical studies have been reported that demonstrate safety of turmeric extracts or formulations over a longer supplementation period. To address this issue, a complete safety program has been established for the novel colloidal suspension TPG, including a preclinical set of toxicological studies and a human clinical safety trial on healthy volunteers, which demonstrated that this turmeric colloidal suspension is safe at a high dosage of 1000 mg/d for ≤5 wk of supplementation (58, K Phipps, S Brinet, H Chevallier, unpublished results, 2021).

Despite numerous discoveries, a limitation of the current study was the number of evaluated outcomes that could increase the risk of type I error, and therefore lead to possible false positives during the statistical analysis. However, the use of the Tukey method adjusting for 10 group comparisons when only 7 comparisons are studied (only STE and TPG compared with all) was quite conservative, and must be highlighted. Another limitation was that some statistical models could not be performed because <15% (45/308) of unconjugated curcuminoid plasma concentrations only were different from zero. In addition, we observed a lot of missing data (minimum ≈50%) for the calculation of AUC 0–infinity (data not shown) because it is necessary to have ≥3 values from the time to peak value (Tmax included), and a descendant phase after Tmax. It should be noted that relative bioavailability, *t*_1/2_, and λz parameters are linked to AUC 0–infinity (use of these parameters during their calculations), and therefore consequent data might be missing in the statistical analysis of these parameters (see considered data in **Supplemental Table 6**). The absolute values of other studies cannot be compared with the results of this study due to differences in participants, analytical methods, study design, and administration of the product.

In conclusion, enhanced bioavailability of turmeric curcuminoids is desirable in view of their encouraging various health benefits but challenged by their poor absorption and rapid metabolism. This study is unique with respect to the relatively large number of participants (*n* = 30), quantitative analysis of multiple metabolites (i.e., 15), and comparison of 5 formulations of turmeric extract at their recommended dosages. Not all curcuminoid formulations were found to deliver equivalent pharmacokinetic profiles. Nevertheless, the new colloidal suspension TPG was revealed to be particularly promising in that 300 mg was sufficient to demonstrate bioequivalence to >1500 mg of the standard turmeric extract STE.

## Supplementary Material

nxab087_Supplemental_File

## Data Availability

Data described in the manuscript will be made available upon request pending application and approval.

## Notes

This work was funded by Naturex SA. The study was performed at Biofortis, Nantes, France.

Supplemental Methods, Supplemental Tables 1–6, and Supplemental References are available from the “Supplementary data” link in the online posting of the article and from the same link in the online table of contents at https://academic.oup.com/jn.

Author disclosures: PF-B, AM, JL, and MT are employees of Naturex SA. Naturex is an international group specializing in plant extraction and production of natural ingredients for food, nutrition and health, and personal care sectors. RBvB is a research collaborator of Naturex. Data management and statistical analysis were exclusively performed by the authors affiliated to Biofortis. All other authors report no conflicts of interest.

Abbreviations used: BDMC, bisdemethoxycurcumin; Cmax, peak plasma concentration; DMC, desmethoxycurcumin; HHC, hexahydrocurcumin; ITT, intent-to-treat; NOV, liquid micellar turmeric extract formulation; PHYT, phytosome turmeric extract formulation; STE, standard turmeric extract containing 95% curcuminoids; *t*_1/2_, half-life; TEP, piperine-curcuminoids combination product; THC, tetrahydrocurcumin; Tmax, time to maximum concentration; TPG, Turmipure Gold turmeric extract formulation; UHPLC, ultra-high-pressure liquid chromatography; λz, terminal elimination rate constant.
