# Supplementary material for: Pharmacokinetics of a Single Dose of Turmeric Curcuminoids Depends on Formulation: Results of a Human Crossover Study
Source: J Nutr. 2021 Apr 20;151(7):1802–16. doi: 10.1093/jn/nxab087 (PMC8245892; doi:10.1093/jn/nxab087)
Supplement: nxab087_Supplemental_File [file nxab087_supplemental_file.docx]

**Supplemental Methods**

*Chemicals and Study materials.* Curcumin (USP #1151855), Demethoxycurcumin (USP #1173100), Bisdemethoxycurcumin (USP #1075305) analytical standards were purchased from Merck (St Quentin Fallavier, France), Hexahydrocurcumin (#82559) and Tetrahydrocurcumin (#82665) were purchased from Phytolab (Vestenbergsgreuth, Germany), Curcumin-d6 (#C838502), Demethoxycurcumin-d7 (#D230817), Bisdemethoxycurcumin-d8 (#B425802), Hexahydrocurcumin-d6 (#H281277) and Tetrahydrocurcumin-d6 (#T293387) were purchased from Toronto Research Chemicals (TRC, North York, Canada). ULC-MS Acetonitrile 0.1% formic acid and ULC-MS water 0.1% formic acid solvents used as eluent for HPLC/MS/MS as well as ULC-MS Methanol and ULS-MS Acetonitrile were purchased from Biosolve (Dieuze, France). Merck (St Quentin Fallavier, France) supplied citric acid (#27109).

*Extraction of curcuminoids.* After deconjugation, plasma underwent protein precipitation, solid-phase extraction and filtration. Briefly, 100 µL of deconjugated plasma or untreated plasma was loaded and mixed with 500 µL of cold methanol/acetonitrile (15:85; v/v) containing internal standards [d_6_]-curcumin, [d_7_]-demethoxycurcumin, [d_8_]-bisdemethoxycurcumin-, [d_6_]- tetrahydrocurcumin, and [d_6_]-hexahydrocurcumin at 50 ng.mL^-1^ on each solid phase extraction cartridge. Curcuminoids were eluted with 200 µL of water/acetonitrile (20:80; v/v) prior to analysis using UHPLC-MS/MS.

*Standardization of curcumin dietary supplements.* For the quantification of curcuminoids in the study supplements, the contents of 10 capsules of each were mixed individually, and 20 mg were weighed into a 50 mL volumetric flask and extracted with ethyl acetate for 5 min using an ultrasonic bath containing ice. The solution was filtered and analyzed using UHPLC-MS/MS for the quantification of curcumin, DMC and BDMC. Curcuminoids were quantified on an Agilent 1200 system (Santa Clara, CA, United States) equipped with a binary pump, autosampler (maintained at 15°C) and DAD detector. Separation was performed on Zorbax Eclipse-C18 (150 x 4.6 mm, 5µm) at 1 mL/min flow in isocratic elution mode (1mg.mL^-1^ citric acid in water / acetonitrile (60:40; v/v) that allowed the resolution of the three curcuminoids detected at 420 nm. The column temperature was 25 °C, and the injection volume was 2 µL.

*UHPLC-MS/MS of curcuminoids in plasma.*  Unconjugated and conjugated curcumin, DMC, BDMC, THC, and HHC concentrations were measured in plasma using UHPLC-MS/MS on an Agilent (Santa Clara, CA, United States) 6420 triple quadrupole mass spectrometer equipped with a 1290 UHPLC system and electrospray. Chromatographic separation was obtained using a Waters BEH-C_18_ column (2.1 x 100 mm, 1.7 µm) fitted with a precolumn cartridge containing the same stationary phase. The column temperature was 35 °C, and the injection volume was 2 µL. The mobile phase consisted of a linear gradient from solvent A (water 0.1% formic acid) to solvent B (acetonitrile 0.1% formic acid) at a flow rate of 400 µL/min as follows: 40% to 60% B over 3 min, 60% to 80% B from 3 to 4 min, and 80% to 100% B from 4 to 6 min. The column re-equilibration time was 4 min between analyses.

Mass spectrometric detection utilized selected reaction monitoring (SRM) with polarity switching and a dwell time of 50 ms per transition. The quantifier SRM transitions were *m/z* 369.1 to *m/z* 177.1 for curcumin (positive ion), *m/z* 339.1 to *m/z* 177.1 for DMC (positive ion), *m/z* 309.1 to *m/z* 147.0 for BDMC (positive ion), *m/z* 371.1 to *m/z* 235.1 for THC (negative ion), *m/z* 373.1 to *m/z* 193.1 for HHC (negative ion). Two additional transitions for each compound were used as qualifiers (see **Supplemental Table 2** for qualifier SRM transitions and for the internal standard SRM transitions).

A stock solution was prepared in methanol containing each curcuminoid standard at 250 µg.mL^-1^, and aliquots were diluted using methanol to prepare 6 calibration solutions from 10 to 600 ng.mL^-1^. The calibration curves were linear (r = 0.99) for all components. The method was validated based on linearity, specificity, precision, accuracy, and robustness according to ICH guidelines (1) (**Supplemental Table 3**). Quality control samples (blank human plasma spiked with known curcumin concentration) were analyzed every 10 samples, and a standard control at the Limit of Quantification (LOQ) was measured at the beginning and at the end of the sequence. The limit of detection (LOD defined as signal to noise of 3) was 0.61 ng.mL^-1^ for curcumin, 0.94 ng.mL^-1^ for DMC, 0.45 ng.mL^-1^ for BDMC, 0.96 ng.mL^-1^ for THC, and 3.44 ng/mL^-1^ for HHC, and the LOQ values were defined as signal to noise of 10.

*Statistical analyses.* All studied AUC were total AUC, i.e. area under the curve above the level of zero. AUC between 0 and 24h calculations were performed using the linear-log trapezoidal rule. The dose-normalized AUC 0-24h and Cmax were calculated by dividing observed AUC 0-24h and Cmax by the corresponding curcuminoids dosage of each formulation. Dose-normalized AUC were analyzed using a mixed model for repeated measurements with product, visit, and baseline values as fixed effects, and subject as a random effect (Y = Product + Visit + Baseline + Subject_random_). Other secondary outcomes, except relative bioavailability, were analyzed using the same calculation model. Relative bioavailabilities were calculated by dividing the dose-normalized AUC 0-24h or AUC 0-8h of the different formulations (TPG, TEP, PHYT or NOV) by the dose-normalized AUC 0-24h or AUC 0-8h of the reference product (STE). Relative bioavailabilities were analyzed using a mixed model for repeated measurements with product and visit values as fixed effects and subject as a random effect (Y = Product + Visit + Subject_random_). Possible sex effect was also investigated with the addition of sex and product-sex interaction as fixed effects in the mixed model for repeated measurements (Y = Product + Visit + Sex + Product*Sex+ Baseline + Subject_random_). Multiple pairwise comparisons between products, performed only in the case of significant product effect, used Tukey adjustment. Although the Tukey Method was adjusted for 10 comparisons (all comparisons possible), only 7 comparisons (comparisons presented in this study) were actually implemented.

For all models, in case of significant visit effect (*P* < 0.05), only the first visit was studied to assess the product effect. To note, all values below the LOD were replaced by 0 (2).

There was no specific treatment of missing data according to the studied population. There was no imputation. In mixed models, missing data for some visits were not replaced but handled by maximum likelihood estimation (3). For kinetics, missing data handling was performed as follows:

- If more than 2 values or 2 consecutive values were missing in the kinetics, the AUC calculation could not be performed and the kinetics was considered as missing in the statistical analyses (no missing data replacement was performed);
- If a data was missing at T-10 time-point, the AUC calculation could not be performed and the kinetics was considered as missing in the statistical analyses (no missing data replacement will be performed);
- If a value (except the baseline value and the value at the last time-point) was missing in the kinetics, it was replaced by the value obtained using the CopyMean method developed by Genolini et *al.* (4). This method has been applied to 1/1650 kinetic sample only during the supplementation of piperine-curcuminoid combination TEP (blood sampling was not performed at T45min due to oversight);
- If a value at the last time-point (T24h) of the kinetics was missing, no missing data replacement was performed. This specific data handling has been applied to 1/1650 kinetic sample only during the supplementation of the phytosome formulation PHYT (blood sampling was not performed due to difficulties of sampling), affecting all the corresponding kinetic parameters over 0-24h (one data set missing for PHYT, N = 29).

In case of non-complete kinetics after missing data handling, the AUC could not be calculated (e.g. the PHYT case presented above).

**Supplemental Tables**

**Supplemental Table 1**. Inclusion/Exclusion criteria

| **Inclusion criteria** | **Exclusion criteria** |
| --- | --- |
| - Age from 18 and 45 years | - Metabolic or endocrine disorder such as diabetes or hyper/hypo thyroidism |
| - BMI from 19 to 25 kg/m² | - Severe chronic disease such as cancer, HIV, renal failure, hepatic or biliary disorder or disease, chronic inflammatory digestive disease, chronic pulmonary disease, arthritis |
| - Weight stable, within ± 3kg in the last three months | - Irritable bowel syndrome |
| - With routine blood chemistry values within normal ranges | - Current disease states that are contraindicated with dietary supplementation: chronic diarrhea, constipation or abdominal pain, chronic laxatives use |
| - For women: Non-menopausal with the same reliable contraception for at least 3 cycles before and agreeing to maintain it during the entire study (condom with spermicidal gel and estrogen/progestin combination contraception accepted) or menopausal without or with hormone replacement therapy (estrogenic replacement therapy initiated less than 3 months excluded) | - Pathology which could affect the study results or expose the subject to an additional risk according to the investigator |
| - Non-smoking or with tobacco consumption ≤ 5 cigarettes / day and agreeing not to smoke during all experimental sessions (V1 to V5) | - Recent gastroenteritis or food borne illness such as confirmed food poisoning (less than 1 month) |
| - Agreeing not to consume food, drink and condiment containing curcumin, or other curcuminoids (DMC, BDMC) for the whole duration of the study | - Blood donation within 3 months prior to the initial visit or intending to donate within the next 3 months |
| - Good general and mental health in the opinion of the investigator: no clinically significant or relevant abnormalities upon review of medical history or following a physical examination, | - Low venous capital not allowing blood kinetic sampling |
| - Able and willing to participate in the study by complying with the protocol procedures as evidenced by a dated and signed informed consent form | - Known or suspected food allergy, intolerance, or hypersensitivity to any of the study product ingredients and/or of the standard meals (gluten intolerance, celiac disease, etc.) |
| - Affiliated with a social security scheme | - Pregnant, lactating or intending to become pregnant within the next 3 months |
| - Agreeing to be registered as a volunteer in the biomedical research file | - Alcohol or drug dependence |
|  | - Chronic drug treatment (for example anticoagulant, antihypertensive medication, thyroid treatment, asthma treatment, anxiolytic, antidepressant, lipid-lowering treatment, corticosteroids, phlebotonic, veino-tonic, drug with impact on blood circulation) excluding oral and local contraceptives |
|  | - Currently taking (or during the past 3 months) any botanical dietary supplement |
|  | - Consumption of curcumin-containing food supplement or food (turmeric, curry) at least 3 times per week and for 2 weeks prior to testing |
|  | - Currently taking (or during the past 3 months) any prebiotics or probiotics supplementation from food or from dietary supplements |
|  | - Significant change in food habits or physical activity within 3 months of the initial visit or not agreeing to maintain them throughout the study |
|  | - Trying to lose weight with a current or planned specific diet in the next 3 months (hypocaloric, vegan, vegetarian) or exercise regimen |
|  | - History of anorexia nervosa, bulimia or significant eating disorders according to the investigator |
|  | - Consuming more than 3 standard drinks of alcoholic beverages daily for men or 2 daily for women or not agreeing to keep alcohol consumption habits unchanged throughout the study |
|  | - Lifestyle deemed incompatible with the study according to the investigator including a high level of physical activity (defined as more than 10 h of significant physical activity a week, walking excluded) |
|  | - Participation in another clinical trial or within the exclusion period of a previous clinical trial |
|  | - Having received, during the last 12 months, indemnities for a clinical trial higher or equal to 4500 Euros |
|  | - Under legal protection (guardianship, wardship) or deprived of their rights following administrative or judicial decision |
|  | - Presenting a psychological or linguistic incapability to sign the informed consent |
|  | - Impossible to contact in case of emergency |
|  | - Abnormal blood chemistry including glycaemia, GGT, ASAT, ALAT, urea, creatinine, blood count |

ALAT: Alanine aminotransferase; ASAT: aspartate aminotransferase; BDMC: Bisdemethoxycurcumin; DMC: Demethoxycurcumin; GGT: Gamma glutamyl transpeptidase

**Supplemental Table 2.** Turmeric-free meal compositions

|  | **Quantity** | **Measure** | **Energy** | **Protein** | **Carbohydrate** | **Fat** |
| --- | --- | --- | --- | --- | --- | --- |
|  |  |  | *kJ* | *g* | *g* | *g* |
| Dinner (evening before all experimental sessions) |  |  | 2284 | 16.5 | 86.7 | 12.4 |
| Bottle of water | 500 | mL | 0 | 0.0 | 0.0 | 0.0 |
| Can of ravioli, 100% pure beef | 400 | g | 1564 | 14.0 | 56.0 | 8.8 |
| Apple compote cup; no sugar added | 100 | g | 264 | 0.5 | 12.8 | 0.6 |
| Pack of 3 biscuits | 25 | g | 456 | 2.0 | 17.9 | 3.0 |
|  |  |  |  |  |  |  |
| Lunch (day of all experimental sessions) |  |  | 2515 | 19.3 | 67.4 | 25.9 |
| Bottle of water | 500 | mL | 0 | 0 | 0 | 0 |
| Club sandwich roasted chicken and mayonnaise, wholegrain bread | 160 | g | 1795** | 16.8** | 36.7** | 22.3** |
| Apple compote cup; no sugar added | 100 | g | 264 | 0.5 | 12.8 | 0.6 |
| Pack of 3 biscuits | 25 | g | 456 | 2.0 | 17.9 | 3.0 |
|  |  |  |  |  |  |  |
| Afternoon snack (day of all experimental sessions) |  |  | 836 | 2.6 | 31.7 | 7.2 |
| Cartoon orange juice box, organic | 200 | mL | 284 | 1.0 | 16.4 | 0.0 |
| Soft cake with chocolate chips | 30 | g | 552 | 1.6 | 15.3 | 7.2 |
|  |  |  |  |  |  |  |
| Diner (day of all experimental sessions) |  |  | 2120 | 26.1 | 58.1 | 18.6 |
| Bottle of water | 500 | mL | 0.0 | 0.0 | 0.0 | 0.0 |
| Can of blanquette of veal | 400 | g | 1304* | 24.0* | 30.0* | 10.8* |
| Apple compote cup; no sugar added | 100 | g | 264 | 0.5 | 12.8 | 0.6 |
| Soft cake with chocolate chips | 30 | g | 552 | 1.6 | 15.3 | 7.2 |
| Total daily macronutrients intake (day of all experimental sessions) |  |  | 5471 | 47.9 | 157.2 | 51.7 |

*Information obtained on Manufacturer sites and Open food facts site*

** Data given for 100 g and multiplied by 4*

*** Data given for 100 g and multiplied by 1.6*

**Supplemental Table 3.** UHPLC-MS/MS parameters for curcuminoid quantification

| **Compound** | **Precursor ion** | **Product ion** | **Fragmentor** | **Collision energy** | **Polarity** |
| --- | --- | --- | --- | --- | --- |
|  | *m/z* | *m/z* | *V* | *V* |  |
| [d_6_]-Curcumin | 375.2 | 180.0 | 65 | 24 | Positive |
| Curcumin | 369.1 | 177.0 | 120 | 20 | Positive |
|  |  | 145.0 | 120 | 36 | Positive |
|  |  | 117.0 | 120 | 48 | Positive |
| [d_7_]-DMC | 346.1 | 151.0 | 105 | 32 | Positive |
| DMC | 339.1 | 177.0 | 105 | 20 | Positive |
|  |  | 147.0 | 105 | 28 | Positive |
|  |  | 91.1 | 105 | 60 | Positive |
| [d_8_]-BDMC | 317.1 | 151.0 | 80 | 24 | Positive |
| BDMC | 309.1 | 147.0 | 95 | 24 | Positive |
|  |  | 119.0 | 95 | 40 | Positive |
|  |  | 91.1 | 95 | 60 | Positive |
| [d_6_]-HHC | 379.2 | 182.1 | 110 | 20 | Negative |
| HHC | 373.1 | 193.1 | 100 | 12 | Negative |
|  |  | 179.1 | 100 | 16 | Negative |
|  |  | 121.0 | 100 | 56 | Negative |
| [d_6_]-THC | 377.1 | 135.1 | 105 | 60 | Negative |
| THC | 371.1 | 235.1 | 120 | 12 | Negative |
|  |  | 193.1 | 120 | 20 | Negative |
|  |  | 135.0 | 120 | 56 | Negative |

BDMC: Bisdemethoxycurcumin; DMC: Demethoxycurcumin; HHC: Hexahydroxycurcumin; THC: Tetrahydrocurcumin

**Supplemental Table 4.** UHPLC-MS/MS assay validation

| **Compound** | **LOD/LOQ** | **Precision (Intra-day)**  **Conc. vs RSD** | **Precision (Inter-day)**  **Conc. vs RSD** | **Accuracy**  **Level vs Recovery** |
| --- | --- | --- | --- | --- |
|  | *ng.mL^-1^* | *ng.mL^-1^* / *%* | | |
| Curcumin | 0.61 / 1.85 | 10 / 4.10  600 / 2.26 | 50 / 2.60 | 10 / 107.97  600 / 100.80 |
| DMC | 0.94 / 2.81 | 10 / 4.39  600 / 3.73 | 50 / 2.75 | 10 / 97.28  600 / 95.79 |
| BDMC | 0.45 / 1.36 | 10 / 3.66  600 / 5.72 | 50 / 4.02 | 10 / 96.62  600 / 95.57 |
| HHC | 0.96 / 2.89 | 10 / 5.07  600 / 5.80 | 50 / 3.32 | 10 / 106.51  600 / 82.86 |
| THC | 3.44 /10.33 | 10 / 9.89  600 / 3.45 | 50 / 3.77 | 10 / 94.04  600 / 90.81 |

Precision (*n*=6), RSD%: Relative standard deviation (SD/Average x 100).

BDMC: Bisdemethoxycurcumin; Conc.: Concentration; DMC: Demethoxycurcumin; HHC: Hexahydroxycurcumin; THC: Tetrahydrocurcumin

**Supplemental Table 5.** Pharmacokinetics parameters Visit-effect for each particular metabolite or group of curcuminoids after consumption of a single dose of the turmeric formulations STE, TEP, PHYT, NOV, and TPG by healthy human participants

|  | **AUC 0-8h** | **AUC 0-24h** | **Cmax** | **AUC 0-8h normalized,** | **AUC 0-24h normalized** | **Cmax normalized** | **Tmax** | **Half-life** | **Terminal elimination rate constant** | **Relative bioavailability 0-8h** | **Relative bioavailability 0-24h** |
| --- | --- | --- | --- | --- | --- | --- | --- | --- | --- | --- | --- |
|  |  |  |  |  |  |  |  |  |  |  |  |
| Curcumin | N^1^ | N | N | Y | N | Y | N | N | N | N | N |
| DMC | N | N | N | N | N | N | N | N | N | N | N |
| BDMC | N | N | N | N | N | N | N | N | N | N | N |
| Curcumin *Glucuronide* | N | N | N | N | N | N | N | N | N | N | N |
| THC *Glucuronide* | N | N | N | N | N | N | N | N | N | N | N |
| HHC *Glucuronide* | N | N | N | N | N | N | N | N | N | N | N |
| Curcumin *Sulfate* | Y | Y | Y | Y | Y | Y | N | N | N | N | Y |
| THC *Sulfate* | N | Y | N | Y | Y | Y | Y | N | N | N | N |
| HHC *Sulfate* | N | N | N | N | N | N | N | N | N | N | N |
| Total Parent Curcuminoids^1^ | N | N | N | Y | N | Y | N | N | N | N | N |
| Total Curcumin Metabolites^2^ | N | N | N | N | N | N | N | N | N | N | N |
| Total Curcuminoids^3^ | N | N | N | N | N | N | N | N | N | N | N |

N means that no significant visit effect has been identified; thus data analysis was performed on all visits

Y means that a statistically significant visit effect was detected (p<0.05); thus data analysis was performed on visit 1 only

^1^ Total Parent Curcuminoids (unconjugated) = curcumin + DMC + BDMC.

^2^ Curcumin and all its metabolites = curcumin + curcumin sulfate + curcumin glucuronide + THC + THC sulfate + THC glucuronide + HHC + HHC glucuronide + HHC sulfate.

^3^ Total Curcuminoids (Parents + Reduced & their conjugates) = curcumin + curcumin sulfate + curcumin glucuronide + DMC + DMC sulfate + DMC glucuronide + BDMC + BDMC glucuronide + BDMC sulfate + THC + THC sulfate + THC glucuronide + HHC + HHC glucuronide + HHC sulfate.

BDMC: Bisdemethoxycurcumin; DMC: Demethoxycurcumin; HHC: Hexahydrocurcumin; NOV: Liquid micellar formulation; PHYT: Phytosome formulation; STE: Standard turmeric extract; TEP: Piperine-curcuminoids combination; THC: Tetrahydrocurcumin; TPG: Turmipure Gold^®^ formulation.

**Supplemental Table 6.** Pharmacokinetics parameter size of data sets for each particular metabolite or group of curcuminoids after consumption of a single dose of the turmeric formulations STE, TEP, PHYT, NOV, and TPG by healthy human participants

|  | **Kinetics 0-8h^1^** | **Kinetics 0-24h^2^** | | **Half-life^3^** | | | | | **Terminal elimination rate constant^3^** | | | | | **Relative bioavailability 0-8h^3^** | | | | | **Relative bioavailability 0-24h^3^** | | | | |
| --- | --- | --- | --- | --- | --- | --- | --- | --- | --- | --- | --- | --- | --- | --- | --- | --- | --- | --- | --- | --- | --- | --- | --- |
|  | **All** | **STE, TEP, NOV, TPG** | **PHYT** | **STE** | **TEP** | **PHYT** | **NOV** | **TPG** | **STE** | **TEP** | **PHYT** | **NOV** | **TPG** | **STE** | **TEP** | **PHYT** | **NOV** | **TPG** | **STE** | **TEP** | **PHYT** | **NOV** | **TPG** |
|  |  |  |  |  |  |  |  |  |  |  |  |  |  |  |  |  |  |  |  |  |  |  |  |
| Curcumin | 30 | 30 | 29 | 15 | 14 | 20 | 16 | 16 | 15 | 14 | 20 | 16 | 16 | 24 | 24 | 24 | 24 | 24 | 24 | 24 | 23 | 24 | 24 |
| DMC | 30 | 30 | 29 | 26 | 23 | 17 | 28 | 24 | 26 | 23 | 17 | 28 | 24 | 9 | 9 | 9 | 9 | 9 | 9 | 9 | 9 | 9 | 9 |
| BDMC | 30 | 30 | 29 | 26 | 26 | 26 | 28 | 26 | 26 | 26 | 26 | 28 | 26 | 8 | 8 | 8 | 8 | 8 | 8 | 8 | 8 | 8 | 8 |
| Curcumin *Glucuronide* | 30 | 30 | 29 | 21 | 22 | 7 | 30 | 26 | 21 | 22 | 7 | 30 | 26 | 30 | 30 | 30 | 30 | 30 | 30 | 30 | 29 | 30 | 30 |
| THC *Glucuronide* | 30 | 30 | 29 | 20 | 16 | 18 | 30 | 24 | 20 | 16 | 18 | 30 | 24 | 27 | 27 | 27 | 27 | 27 | 27 | 27 | 26 | 27 | 27 |
| HHC *Glucuronide* | 30 | 30 | 29 | 24 | 24 | 14 | 29 | 27 | 24 | 24 | 14 | 29 | 27 | 30 | 30 | 30 | 30 | 30 | 30 | 30 | 29 | 30 | 30 |
| Curcumin *Sulfate* | 30 | 30 | 29 | 19 | 20 | 13 | 29 | 27 | 19 | 20 | 13 | 29 | 27 | 30 | 30 | 30 | 30 | 30 | 30 | 30 | 29 | 30 | 30 |
| THC *Sulfate* | 30 | 30 | 29 | 21 | 19 | 23 | 17 | 13 | 21 | 19 | 23 | 17 | 13 | 16 | 16 | 16 | 16 | 16 | 16 | 16 | 15 | 16 | 16 |
| HHC *Sulfate* | 30 | 30 | 29 | 25 | 26 | 13 | 30 | 29 | 25 | 26 | 13 | 30 | 29 | 30 | 30 | 30 | 30 | 30 | 30 | 30 | 29 | 30 | 30 |
| Total Parent Curcuminoids^4^ | 30 | 30 | 29 | 16 | 15 | 17 | 16 | 16 | 16 | 15 | 17 | 16 | 16 | 24 | 24 | 24 | 24 | 24 | 24 | 24 | 23 | 24 | 24 |
| Total Curcumin Metabolites^5^ | 30 | 30 | 29 | 21 | 22 | 17 | 30 | 27 | 21 | 22 | 17 | 30 | 27 | 30 | 30 | 30 | 30 | 30 | 30 | 30 | 29 | 30 | 30 |
| Total  Curcuminoids^6^ | 30 | 30 | 29 | 20 | 22 | 16 | 30 | 27 | 20 | 22 | 16 | 30 | 27 | 30 | 30 | 30 | 30 | 30 | 30 | 30 | 29 | 30 | 30 |

^1^ Kinetics 0-8h = AUC 0-8h, AUC 0-8h normalized

^2^ Kinetics 0-24h = AUC 0-24h, AUC 0-24h normalized

^3^ Half-life, Terminal elimination rate constant and Relative bioavailabilities are linked to AUC 0-infinity. Considerable data are missing for these parameters due to the necessity of having at least 3 values from the time to peak value (Tmax included), and a descendant phase after Tmax to calculate AUC 0-infinity.

^4^ Total Parent Curcuminoids (unconjugated) = curcumin + DMC + BDMC.

^5^ Curcumin and all its metabolites = curcumin + curcumin sulfate + curcumin glucuronide + THC + THC sulfate + THC glucuronide + HHC + HHC glucuronide + HHC sulfate.

^6^ Total Curcuminoids (Parents + Reduced & their conjugates) = curcumin + curcumin sulfate + curcumin glucuronide + DMC + DMC sulfate + DMC glucuronide + BDMC + BDMC glucuronide + BDMC sulfate + THC + THC sulfate + THC glucuronide + HHC + HHC glucuronide + HHC sulfate.

BDMC: Bisdemethoxycurcumin; DMC: Demethoxycurcumin; HHC: Hexahydrocurcumin; NOV: Liquid micellar formulation; PHYT: Phytosome formulation; STE: Standard turmeric extract; TEP: Piperine-curcuminoids combination; THC: Tetrahydrocurcumin; TPG: Turmipure Gold^®^ formulation.

**Supplemental References**

1. International Conference On Harmonisation Of Technical Requirements For Registration Of Pharmaceuticals For Human Use - ICH Harmonised Tripartite Guideline, Validation Of Analytical Procedures: Text And Methodology Q2(R1) [Internet]. Available from: https://database.ich.org/sites/default/files/Q2_R1__Guideline.pdf

2. Johnson JR. Methods for Handling Concentration Values Below the Limit of Quantification in PK Studies [Internet]. Available from: https://www.pharmpk.com/

3. Twisk J, De Boer M, De Vente W, Heymans M. Multiple imputation of missing values was not necessary before performing a longitudinal mixed-model analysis. J Clin Epidemiol. Pergamon; 2013;66:1022–8.

4. Genolini C, Écochard R, Jacqmin-Gadda H. Copy Mean: A New Method to Impute Intermittent Missing Values in Longitudinal Studies. Open J Stat. 2013;3:26–40. Available from: http://www.scirp.org/journal/ojs.
